# Global Cancer Nurse's Experiences and Perceptions of Potential Occupational Exposure to Cytotoxic Drugs: Mixed Method Systematic Review With Framework Synthesis

**DOI:** 10.1111/jocn.17488

**Published:** 2024-10-29

**Authors:** Karen Campbell, Janyne Afseth, Margaret Dunham, Maria King, Daniel Dicksit

**Affiliations:** ^1^ Edinburgh Napier University Edinburgh UK; ^2^ Robert Gordon University Aberdeen UK

## Abstract

**Aim:**

To conceptualise experiences and perceptions of cancer nurses' potential for occupational exposure when dealing with cytotoxic drugs (CDs).

**Design:**

A mixed methods systematic review with framework synthesis.

**Methods and Data Sources:**

A literature search was conducted in February 2022 in CINAHL PubMed, Web of Science, Ovid Nursing, and PsycINFO, and it was reported using the PRISMA guidance.

**Results:**

A synthesis of 38 studies revealed new categories of perceived solutions, side effects, and risky behaviour as well as three levels of experience and perception: individual, shared, and cultural, rather than the a priori theory.

**Conclusions:**

The review conclude that individuals espouse safe handling and administration of CDs. Synthesis highlights a complex interplay between self‐reported perception and the observed experience of potential occupational exposure to cytotoxic drugs.

**Implications for Professional Practice:**

The framework synthesis highlights the difference between the perception of espoused practice and the experience of practice. Observation and risk assessment must be used to enhance safe practice. Organisations must take seriously the perception and experience of the adverse effects of administering cytotoxic drugs to support cancer nurses.

**Reporting Method:**

Joanna Briggs Institute's (JBI) methodology for systematic reviews and framework synthesis indexed studies deductively and inductively.

No patient or public contribution.

**Trial Registration:**

PROSPERO: CRD42022289276

**Summary**

- What problem did the study address?
  ○ The perception and experience of handling cytotoxic drugs by cancer nurses translate into future policy and practice.
- What were the main three findings?
  ○ The research is based on self‐reported practice, and solutions focus on education and implementing guidelines. Studies report adverse events, including hair loss, reproductive issues, and cancer. The availability of monitoring and closed‐system devices could inadvertenlty result in less wearing of personal protective equipment.
- To whom will the research have an impact?
  ○ Cancer nurses and health and safety policy.

## 1 Introduction

Cytotoxic drugs are hazardous (Control of Substances Hazardous to Health Regulations (COSHH) 2002; NIOSH 2004). Therefore, occupational exposure to cytotoxic drugs, also known as antineoplastic or chemotherapy drugs, can pose significant safety issues for cancer nurses involved in their handling, preparation, administration, and disposal, regardless of the healthcare setting (Eisenberg and Klein 2021).

Occupational exposure is a reality because cytotoxic drugs, administered by any route, either oral (Lester 2012; Rudnitzki and McMahon 2015), intravenous or intrathecally, can be absorbed through the skin, inhalation, or ingestion (Eisenberg and Klein 2021). Direct contact with the drug or exposure to drug‐contaminated surfaces, equipment, or air can result in absorption into the body. Skin contact is a standard route of exposure particularly when handling contaminated surfaces or during drug administration (Connor and McDiarmid 2006; McDiarmid et al. 2010; Hanafi et al. 2015; Eisenberg 2016; Field, Hughes, and Rowland 2017; Simons and Toland 2017).

The short and longer‐term effects of occupational exposure can increase the risk of cancer, reproductive hazards, skin irritation and sensitisation, and respiratory effects. Reported adverse effects, including carcinogenicity, teratogenicity, and mutagenicity, including chromosomal aberrations that mirror those of cancer patients (Polovich 2004; Connor and McDiarmid 2006; McDiarmid et al. 2010; Hanafi et al. 2015; Eisenberg 2016; Field, Hughes, and Rowland 2017; Simons and Toland 2017; Hu et al. 2023). The design of cytotoxic drugs is to kill or inhibit the growth of cancer cells; they also harm the healthy cells of those cancer nurses delivering treatment if not appropriately handled (Meade, Simons, and Toland 2017; Eisenberg and Klein 2021).

To mitigate these safety issues, healthcare facilities and cancer nurses should follow established standardised education (Coyne et al. 2019), nursing and health and safety guidelines and protocols for the safe handling, preparation, administration, and disposal of cytotoxic drugs (Meade 2014; Coyne et al. 2019; Mathias et al. 2019; Oncology Nursing Society 2019). This hierarchy of control includes wearing appropriate personal protective equipment (PPE), implementing engineering controls (e.g., closed systems devices), using proper techniques for drug preparation and administration, and following proper waste management procedures (Yu 2020; Eisenberg and Klein 2021; Meade, Simons, and Toland 2017). Regular monitoring, evaluation, and education are essential to maintaining a safe working environment for healthcare workers handling cytotoxic drugs but are rarely adhered to (Mathias et al. 2019).

Closed systems are one solution to reducing risk in numerous countries; however, these are currently optional (Yu 2020), and the evidence base for their use needs to be more conclusive (Gurusamy et al. 2018; Health Improvement Scotland 2019). Connor and McDiarmid (2006) and Eisenberg and Klein (2021) highlight the need to explore this potential occupational exposure in the cancer nursing population further.

Other reviews in this field of inquiry have focused on factors influencing safe handling precautions and education (Lin et al. 2019) and patient and staff safety requirements (Coyne et al. 2019). Conducting this systematic review to understand cancer nurses' experiences and perceptions of potential occupational exposure to cytotoxic drugs worldwide gives another contextual lens on this topic, helping to understand the safety and wellbeing of this workforce.

**FIGURE 1:**
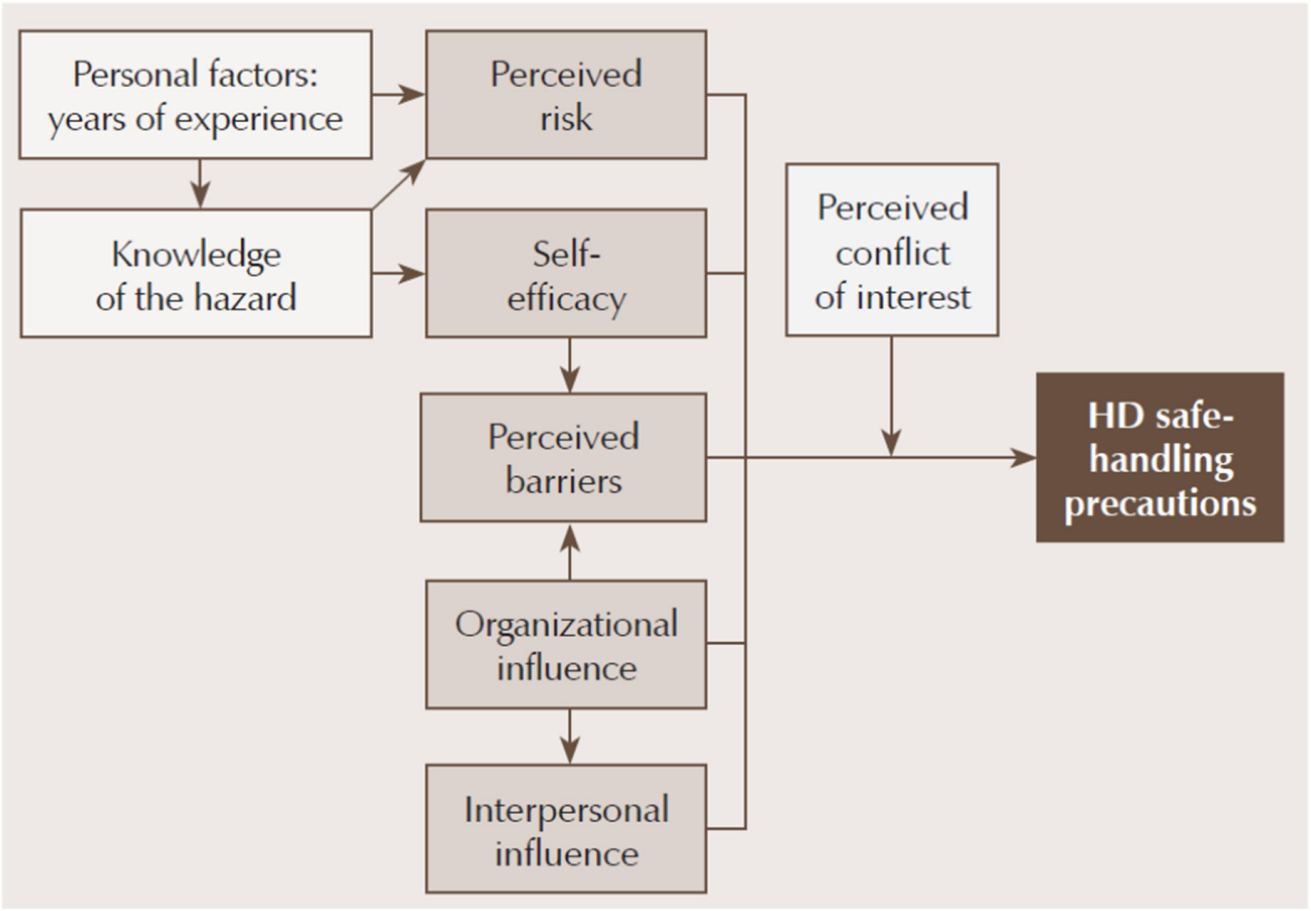
Theoretical framework: factors predicting use of hazardous drug (HD) safe‐handling precautions (Polovich and Clark 2012). From “Predictors of Hearing Protection Use for Hispanic and Non‐Hispanic White Factory Workers,” by D.M. Raymond 3rd, O. Hong, S.L. Lusk, & D.L. Ronis, 2006, Research and Theory for Nursing Practice: An International Journal, 20, p. 129. Copyright 2006 by Springer Publishing Company, LLC. Adapted with permission. [Colour figure can be viewed at wileyonlinelibrary.com]

Due to the often‐emotive nature of this topic, a known theoretical framework for synthesis was applied (Carroll et al. 2013). This approach aids in categorising existing concepts to the priori framework and considers potential new emerging concepts within the existing literature. The framework from Polovich and Clark (2012) (Figure 1) was selected as the priori framework to provide an inductive and deductive synthesis of the evidence base for this review. The theoretical framework has been the only one developed for handling hazardous drugs. This framework provided a complementary approach to the research question posed by allowing the tenets of influencing factors, hypothetically associated with perception and experience of the potential of occupational exposure to cytotoxic drugs, to be integral to the process of the deductive thematic analysis, allowing for themes to emerge direct from using inductive coding (Fereday and Muir‐Cochrane 2006).

The proposed model considers the interaction between the individual and the environment, influencing their behaviour (Polovich and Clark 2012). In Figure 1, knowledge of the hazard is related to perceived risk and self‐efficacy. Higher self‐efficacy in using PPE and positive organisational influences is expected to decrease perceived barriers. Perceived risk, self‐efficacy, perceived barriers, organisational influences, and interpersonal influences are all expected to impact safe handling precautions. Conflict of interest was added as this may be associated with patient needs rather than individual control.

### 1.1 Aim

The study aims to understand cancer nurses' experiences and perceptions of potential occupational exposure to cytotoxic drugs.

## 2 Methodology

### 2.1 Search Methods

For this study, we adhered to the Joanna Briggs Institute (JBI) methodology for systematic reviews and reviewed the cancer nurses' experiences and perceptions of potential exposure to cytotoxic drugs. For a complete set of database searches and results, see *data base searches and results*, Appendix S1. Restricted publication dates were from 2000 until early 2022, and results were limited to the English language only where the database allowed.

The following databases and platforms were searched between the 18th and 24th of February 2022: CINAHL with Full text (EBSCO), PubMed (including Medline and PMC), Web of Science, Ovid Nursing, PsycINFO (EBSCO) using the search terms ‘cancer nurs*’, ‘perception’, ‘experiences’ ‘cytotoxic drugs’, and ‘occupational exposure’. See Appendix S1 for a fuller search strategy. A hand‐search was conducted online in a University Library catalogue, Library Search, and Google Scholar, as well as in cancer and oncology nursing journals, available via subscriptions with full text not indexed in any searched databases. These include the European Journal of Oncology Nursing, Seminars in Oncology Nursing, and Cancer Nursing Practice.

Grey literature searching was undertaken using Google, and the specific organisational websites of the European Oncology Nursing Society, Oncology Nursing Society, and UK Oncology Nursing Society were looked at. In addition, forward and backward citation searches were conducted from the included articles.

The guidelines of The PRISMA 2020 (*Guidelines for reporting systematic reviews* Appendix S2) statement, an updated guideline for reporting systematic reviews, will report the review results (Page et al. 2021). The review protocol is registered (ID CRD42022289276) on The International Prospective Register of Systematic Reviews (PROSPERO).

### 2.2 Inclusion and Exclusion Study Selection

Articles were managed in Endnote, including the removal of duplicates. They were then exported onto Rayyan QCRI for screening. Three reviewers independently screened the titles and abstracts against pre‐defined eligibility criteria. A fourth reviewer resolved disagreements between the reviewers. Where abstracts were unavailable, full‐text articles were obtained, and this review identified 38 studies under the inclusion criteria (Table 1).

**TABLE 1:** Inclusion and exclusion criteria.

	Inclusion	Exclusion
Study type	An empirical article: qualitative, quantitative, or mixed methods or nonexperimental (cohort studies)	Systematic reviews and literature reviews
Setting	All care settings in which cytotoxic drugs are administered	No administration or handling of cytotoxic drugs
Population	Cancer nurses handling cytotoxic drugs during preparation, administration, disposal, and handling patient excreta	Other health care professionals than nursing, for example, pharmacy and nursing assistants. Also, studies that showed nurses comprise less than 20% of the population
Context	Potential occupational exposure when handling cytotoxic drugs	Not handling cytotoxic drugs. Handling of antibiotics, immunotherapy, and /or antibody therapy
Concept	Reporting factors associated with perception and experience	Not reporting factors influencing associated with perception and experience
Publication type	Primary research studies published in peer‐reviewed journals	Conference abstracts, book chapters, reviews, commentaries, editorials, and study protocols
Language	Published in English	Published in other languages other than English
Date	Published since 2000, just before and after the control of substances hazardous to health	Published before 2001

### 2.3 Data Evaluation

#### 2.3.1 Quality Assurance

Two reviewers independently assessed included studies using quality scoring for methodological content (adapted from Hawker et al. 2002). The assessment included relevance to the research question, the data source, and the study type. Subsequently, each paper was assigned a score ranking, noted in the findings table (*Study characteristics* Appendix S3), with any specific factors, acknowledging the heterogeneity of the studies and possible methodological limitations, including where some of the quality criteria were not applicable. The completed quality appraisal did not impact the study's eligibility to be included and aimed to generate an overall quality assessment.

### 2.4 Data Extraction and Synthesis of the Included Studies

**FIGURE 2:**
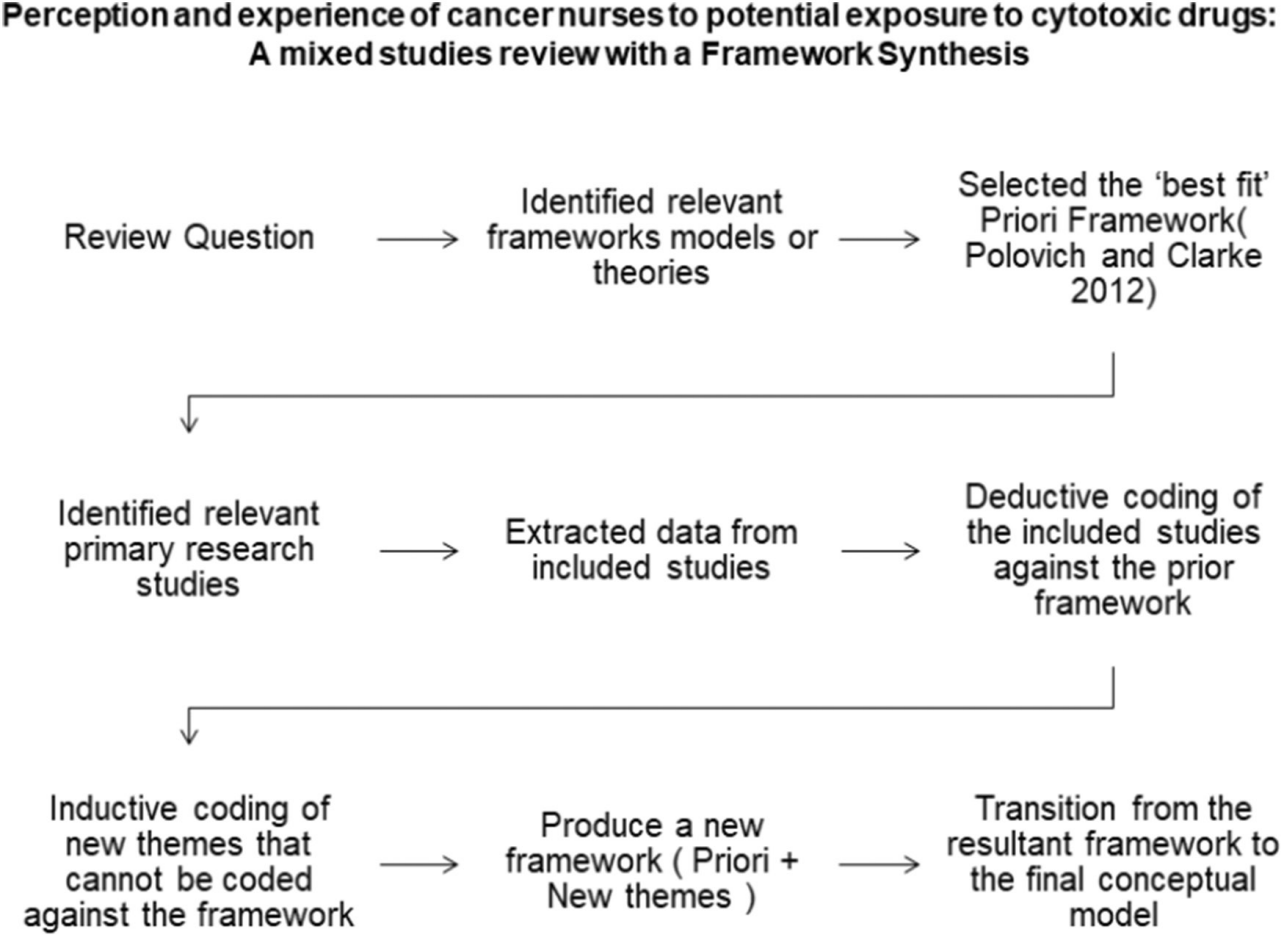
Framework synthesis process (Granikov et al. 2022).

A framework synthesis (Gale et al. 2013) was completed to categorise the studies by coding, indexing, and theming against the priori framework (Polovich and Clark 2012) with factors: knowledge of the hazard, perceived risk, self‐efficacy, perceived barriers, organisational influence, interpersonal influence, personal factors, and conflict of interest (Figure 2).

### 2.5 Findings

**FIGURE 3:**
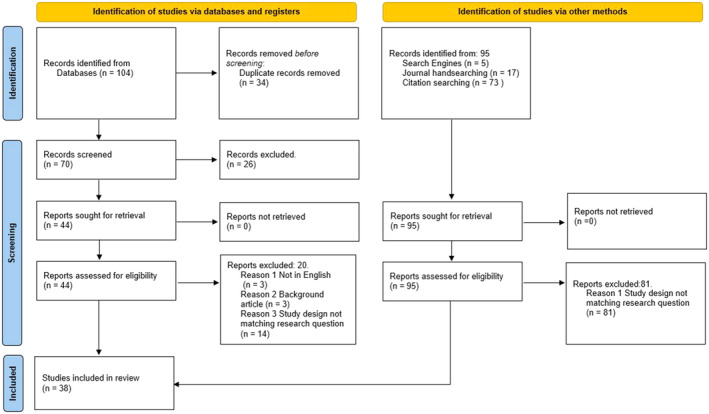
PRISMA 2020 diagram (Page et al. 2021). [Colour figure can be viewed at wileyonlinelibrary.com]

The initial search provided 179 studies, of which 34 were duplicates. One hundred and seven records were excluded at the title and abstract review stage. This review includes 38 studies reported in 41 journal articles, with Graeve, McGovern, Alexander, et al. (2017), Graeve, McGovern, Arnold, et al. (2017), Soheili, Jokar, et al. (2021), Soheili et al. (2021a), and Soheili et al. (2021b) covering the same study population but different publications (Figure 3).

### 2.6 Study Characteristics

Twenty‐six studies were quantitative, five were qualitative, and seven were mixed methods. The articles were then organised into a data extraction sheet (*Study characteristics* Appendix S3).

### 2.7 Country of Origin

Eight studies were from the USA (Callahan et al. 2016; Colvin, Karius, and Albert 2016; DeJoy et al. 2017; Graeve, McGovern, Alexander, et al. 2017; Graeve, McGovern, Arnold, et al. 2017; He et al. 2017; Polovich and Clark 2012; Silver, Steege, and Boiano 2016); Seven studies were conducted in Turkey (Baykal, Seren, and Sokmen 2009; Çınar and Karadakovan 2022; Kosgeroglu et al. 2006; Kutlutürkan and Kırca 2022; Topçu and Beşer 2017; Tuna and Baykal 2017; Turk et al. 2004); five studies were from Iran (Alehashem and Baniasadi 2018; Hanafi et al. 2015; Orujlu et al. 2016; Shahrasbi et al. 2014; Soheili, Jokar, et al. 2021; Soheili et al. 2021a; Soheili et al. 2021b); three studies are from the UK (Simons and Toland 2017, 2019; Verity et al. 2008) and two studies in Brazil (Batista et al. 2022; Borges, Silvino, and dos Santos 2015). The other studies were from Ethiopia (Asefa et al. 2021), Egypt (Mahdy, Rahman, and Hassan 2017), Greece (Constantinidis et al. 2011), Israel (Ben‐Ami et al. 2001), France (Benoist et al. 2022), Taiwan (Chen, Lu, and Lee 2016), Nigeria (Nwagbo et al. 2017), Canada (Hon, Teschke, and Shen 2015), South Korea (Kim et al. 2019), Jordan (Abu Sharour et al. 2021), Pakistan (Khan, Khowaja, and Ali 2012), Thailand (Srisintorn et al. 2021), Spain (Bernabeu‐Martínez et al. 2021), and Cyprus (Kyprianou et al. 2010), with one study from each country.

### 2.8 Theoretical Frameworks

Seven studies utilised the theoretical framework—Factors Predicting the Use of Hazardous Drugs (HD) Safe Handling Precautions (Abu Sharour et al. 2021; Callahan et al. 2016; Graeve, McGovern, Alexander, et al. 2017; Graeve, McGovern, Arnold, et al. 2017; He et al. 2017; Mahdy, Rahman, and Hassan 2017; Polovich and Clark 2012; Srisintorn et al. 2021). Topçu and Beşer (2017) utilised the Health Belief Model: perceived sensitivity, perceived seriousness, perceived benefits, perceived barriers, and “cues to actions.” Ben‐Ami et al. (2001) and Nwagbo et al. (2017) studies utilised the Health Belief Model and its extended form of Protection Motivation Theory (PMT). The PMT model assumes that engaging in specific health behaviours is a direct function of a person's motivation to protect oneself: perceived susceptibility, severity, perceived benefits, perceived barriers, and self‐efficacy.

### 2.9 Study Measurement Tools

Thirty‐three studies used surveys with 22 being adapted questionnaire (Abu Sharour et al. 2021; Ben‐Ami et al. 2001; Benoist et al. 2022; Borges, Silvino, and dos Santos 2015; Callahan et al. 2016; Colvin, Karius, and Albert 2016; Graeve, McGovern, Alexander, et al. 2017; Graeve, McGovern, Arnold, et al. 2017; He et al. 2017; Hon, Teschke, and Shen 2015; Khan, Khowaja, and Ali 2012; Kim et al. 2019; Kosgeroglu et al. 2005; Kutlutürkan and Kırca 2022; Kyprianou et al. 2010; Mahdy, Rahman, and Hassan 2017; Orujlu et al. 2016; Polovich and Clark 2012; Shahrasbi et al. 2014; Silver, Steege, and Boiano 2016; Srisintorn et al. 2021; Turk et al. 2004; Verity et al. 2008) or a newly developed questionnaire (Alehashem and Baniasadi 2018; Baykal, Seren, and Sokmen 2009; Bernabeu‐Martínez et al. 2021; Çınar and Karadakovan 2022; Constantinidis et al. 2011; DeJoy et al. 2017; Hanafi et al. 2017; Nwagbo et al. 2017; Simons and Toland 2017, 2019).

Six studies conducted face‐to‐face or telephone interviews (Asefa et al. 2021; Benoist et al. 2022; Chen, Lu, and Lee 2016; Polovich and Clark 2012; Soheili, Jokar, et al. 2021; Soheili et al. 2021a; Soheili et al. 2021b; Topçu and Beşer 2017). Six observation studies were conducted after surveys to monitor the practice of safe handling of cytotoxic drugs (Ben‐Ami et al. 2001; Chen, Lu, and Lee 2016; Colvin, Karius, and Albert 2016; Hanafi et al. 2017; Kosgeroglu et al. 2006; Shahrasbi et al. 2014).

The mapping of all included articles in the review to the priori framework aligned with the methodology approach of framework synthesis (Table 2). The mapping visually represents where each paper aligns with the framework. In addition, it briefly highlights the research priority under investigation, reporting a decrease in focus from left to right.

**TABLE 2:** Mapping to the original priori framework.

Authors study	Research Interest of variables of perception and experience in decreasing order											
	Safe handling precautions	Perceived solutions	Knowledge of hazard	Organisational influence	Personal factors	Side effects	Perceived risks	Perceived barriers	Self‐efficacy	Perceived conflict of interest	Risky behaviour	Interpersonal factors
Abu Sharour et al. (2021) Jordan	X		X	X	X		X	X	X	X		X
Alehashem and Baniasadi (2018) Iran	X	X	X		X							
Asefa et al. (2021) Ethiopia	X	X	X	X	X			X				
Batista et al. (2021) Brazil	X		X			X						
Baykal, Seren, and Sokmen (2009) Turkey	X	X	X	X		X	X				X	
Ben‐Ami et al. (2001) Israel	X	X	X			X	X	X	X		X	
Benoist et al. (2022) France	X	X	X	X	X	X	X					
Bernabeu‐Martínez et al. (2021) Spain							X					
Borges, Silvino, and dos Santos (2015) Brazil	X		X			X						
Callahan et al. (2016) USA	X	X	X	X	X		X	X	X	X		X
Chen, Lu, and Lee (2016) Taiwan	X	X		X	X		X	X	X	X		
Çınar and Karadakovan (2022) Turkey	X	X						X				
Colvin, Karius, and Albert (2016) USA	X	X							X			
Constantinidis et al. (2011) Greece	X	X				X		X				
DeJoy et al. (2017) USA	X	X		X	X		X					
Graeve, McGovern, Alexander, et al. (2017), Graeve, McGovern, Arnold, et al. (2017) USA	X	X	X		X		X	X	X	X	X	X
Hanafi et al. (2017) Iran	X	X	X		X	X					X	
He et al. (2017) USA	X				X	X						X
Hon, Teschke, and Shen (2015) Canada	X		X				X		X			
Khan, Khowaja, and Ali (2012) Pakistan		X	X				X			X		
Kim et al. (2019) South Korea	X			X	X			X	X			
Kosgeroglu et al. (2006) Turkey	X	X	X	X	X				X		X	
Kutlutürkan and Kırca (2022) Turkey				X		X			X			
Kyprianou et al. (2010) Cyprus	X	X	X	X	X	X	X					
Mahdy, Rahman, and Hassan (2017) Egypt	X	X		X		X		X				
Nwagbo et al. (2017) Nigeria	X	X	X		X							
Orujlu et al. (2016) Iran	X	X	X	X	X	X		X				
Polovich and Clark (2012) USA	X	X	X	X	X		X	X	X	X		X
Shahrasbi et al. (2014) Iran	X	X	X	X		X						
Silver, Steege, and Boiano (2016) USA	X	X		X								
Simons and Toland (2017) UK	X					X						
Simons and Toland (2019) UK	X	X	X			X	X					
Soheili, Jokar, et al. (2021), Soheili et al. (2021a), Soheili et al. (2021b) Iran	X			X		X	X		X			
Srisintorn et al. (2021) Thailand	X	X	X	X	X		X	X	X	X		X
Topçu and Beşer (2017) Turkey	X	X		X		X	X	X				
Tuna and Baykal (2017) Turkey	X	X		X		X						
Turk et al. (2004) Turkey	X	X	X		X	X					X	
Verity et al. (2008) UK		X	X		X		X					
Total	34	28	23	20	19	19	18	14	13	7	6	6

## 3 Narrative Summary of the Mapping to Priori Framework

### 3.1 Personal Factors

Personal factors were associated with the demography of the population and the level of nursing chemotherapy experience. Seven studies reported significant correlations when comparing the demographical factors such as education, age, and work experience (Alehashem and Baniasadi 2018; Asefa et al. 2021; Chen, Lu, and Lee 2016; DeJoy et al. 2017; Kim et al. 2019; Srisintorn et al. 2021; Abu Sharour et al. 2021). Eight studies compared the demography with other variables that reported no significant differences (Benoist et al. 2022; Graeve, McGovern, Alexander, et al. 2017; Graeve, McGovern, Arnold, et al. 2017; Hanafi et al. 2015; Kosgeroglu et al. 2006; Kyprianou et al. 2010; Polovich and Clark 2012; Turk et al. 2004; Verity et al. 2008).

### 3.2 Level of Knowledge of Hazards

Seven studies reported a high level of knowledge of occupational exposure among their participants (Ben‐Ami et al. 2001; Borges et al. 2015; Callahan et al. 2016; Graeve, McGovern, Alexander, et al. 2017; Graeve, McGovern, Arnold, et al. 2017; Hon, Teschke, and Shen 2015; Nwagbo et al. 2017; Orujlu et al. 2016; Srisintorn et al. 2021). Seven studies reported an adequate level of knowledge of occupational exposure amongst their participants (Alehashem and Baniasadi 2018; Batista et al. 2021; Benoist et al. 2022; Hanafi et al. 2017; Kyprianou et al. 2010; Polovich and Clark 2012; Shahrasbi et al. 2014). Seven studies reported a lack of knowledge of occupational exposure amongst their participants (Abu Sharour et al. 2021; Asefa et al. 2021; Baykal, Seren, and Sokmen 2009; Khan, Khowaja, and Ali 2012; Simons and Toland 2019; Turk et al. 2004; Verity et al. 2008). Kosgeroglu et al. (2006) was the only study that referred to nurses being aware but then needing to apply the knowledge to practice.

### 3.3 Perceived Risks

In measuring perceived risk, nine studies reported that their participants had a high perceived risk (Abu Sharour et al. 2021; Callahan et al. 2016; DeJoy et al. 2017; Kyprianou et al. 2010; Polovich and Clark 2012; Simons and Toland 2019; Soheili et al. 2021a; Srisintorn et al. 2021; Verity et al. 2008) and conversely five studies reported a low perceived risk from their participants (Benoist et al. 2022; Ben‐Ami et al. 2001; Chen, Lu, and Lee 2016; Khan, Khowaja, and Ali 2012; and Topçu and Beşer 2017). Chen, Lu, and Lee (2016) further reported the perceived risk of toxicity as ‘encapsulated’ and ‘well‐diluted’ by the pharmacist before reaching them for administration. Topçu and Beşer (2017) noted that low perceived risk was associated with ‘contamination is impossible’ when using closed systems.

Hon, Teschke, and Shen (2015) reported a statistically significant (*p* = 0.002) difference in perception of the risk when pharmacists downplayed preparing the drugs compared to nurses administering the drugs. Graeve, McGovern, Alexander, et al. (2017) and Graeve, McGovern, Arnold, et al. (2017) showed a statistically significant increase in self‐perceived risk using pre and post‐survey after training and contamination swabbing result intervention study.

There were various consequences of having a high perceived risk. Baykal, Seren, and Sokmen (2009) reported that a perception of high risk made the nurses *not* want to work in the oncology department due to perceived health concerns. Conversely, Polovich and Clark (2012) correlated higher perceived risks to a better safety climate in the nurses' unit.

Bernabeu‐Martínez et al. (2021) examined the perceived risk of their participants by asking about each practical stage of the administration process. The perceived risk of the participants was lowest for transporting the CD to the place of administration. The highest risk was associated with accidental exposure during connection and disconnection of infusion lines and areas around the spike, where there is the risk of exposure by drops and spills, tears, or inadequate connection. Bernabeu‐Martínez et al. (2021) stated that administration followed by waste management was perceived as the highest activity in the potential for occupational exposure. The intravesical installation presented the most significant risk, followed by premade bolus / intermuscular, with infusional and ocular administration being identified as the least potential for occupational exposure. The nurse reported the risk of exposure to be higher and associated with the administration phase of the process.

### 3.4 Self‐Efficacy

Self‐efficacy was perceived to be linked to the ability to perform self‐measures over time, contributing to their health (Ben‐Ami et al. 2001). Callahan et al. (2016) stated a high level of self‐efficacy in their study, whereas Abu Sharour et al. (2021) and Polovich and Clark (2012) noted a medium level of self‐efficacy. A perceived influencing factor in self‐efficacy was clinical knowledge and skill reported by Hon, Teschke, and Shen (2015), Kutlutürkan and Kırca (2022), and Soheili et al. (2021a). Five studies reported self‐efficacy and adherence to PPE guidance in reducing exposure to CDs (Chen, Lu, and Lee 2016; Graeve, McGovern, Alexander, et al. 2017; Graeve, McGovern, Arnold, et al. 2017; Kim et al. 2019; Kosgeroglu et al. 2006; Srisintorn et al. 2021). Double gloving during the disconnection of the IV line and washing hands after the administration of chemotherapy were described as safety measures to reduce exposure by Colvin, Karius, and Albert (2016).

### 3.5 Perceived Barriers

Orujlu et al. (2008) reported that using PPE during waste disposal and cleaning spills was less than other activities in the study. Six studies stated that a lack of wearing PPE was due to discomfort, work pressures, or availability (Ben‐Ami et al. 2001; Callahan et al. 2016; Graeve, McGovern, Alexander, et al. 2017; Graeve, McGovern, Arnold, et al. 2017; Kim et al. 2019; Mahdy, Rahman, and Hassan 2017; Srisintorn et al. 2021). Asefa et al. (2021) stated that PPE was not required, and Chen, Lu, and Lee (2016) said it was due to a barrier due to cost implications. Constantinidis et al. (2011) and Topçu and Beşer (2017) cited a lack of training as a barrier. The number of nurses, lack of payments, extra leave, and psychological support were perceived barriers by Çınar and Karadakovan (2022). Polovich and Clark (2012) study reported the low perceived barriers associated with higher safe handling.

### 3.6 Organisational Influence

Four studies stated that a lack of training for administrating cytotoxic drugs has been identified as influencing work safety climate in several studies (Asefa et al. 2021; Benoist et al. 2022; Kutlutürkan and Kırca 2022; Shahrasbi et al. 2014). Chen, Lu, and Lee (2016), Kim et al. (2019), and Tuna and Baykal (2017) all cited that cost‐cutting measures and insufficient PPE availability make the environment unsafe. Six studies highlighted that the perceived safe climate in the workplace improved the usage of PPE (Abu Sharour et al. 2021; Callahan et al. 2016; DeJoy et al. 2017; Kim et al. 2019; Polovich and Clark 2012; Srisintorn et al. 2021).

Six studies described the nurses' perception of working conditions and environment as longer working hours (Kosgeroglu et al. 2006; Orujluo et al. 2008; Baykal, Seren, and Sokmen 2009; Topçu and Beşer 2017; Tuna and Baykal 2017; Kutlutürkan and Kırca 2022), whereas Kyprianou et al. (2010) and Mahdy, Rahman, and Hassan (2017) described high workloads. Four studies suggested that lower pay and lack of overtime payments lead to burnout and emotional disturbances among nurses linked to a variety of adverse outcomes in healthcare, including worker errors and injuries (DeJoy et al. 2017; Silver, Steege, and Boiano 2016; Orujluo et al. 2008; Soheili, Jokar, et al. 2021; Soheili et al. 2021a; Soheili et al. 2021b).

Soheili et al. (2021a) identified organisational influences that could include inadequate ventilation, lighting, and noise reduction. Chen, Lu, and Lee's (2016) study perceived that the higher role status in the organisation's pay created resistance to being transferred, even if pregnant.

Chen, Lu, and Lee (2016) reported a cultural difference when observing nurses administering CDs where the patients' needs came first, and PPE was time‐consuming and interrupted their schedule. The nurses stated in this study that their expertise in administering CDs meant that they would not be exposed to PPE when opting out.

### 3.7 Interpersonal Influences

Callahan et al. (2016) and Polovich and Clark (2012) reported strong interpersonal influence by nurses, which resulted in using precautions while handling CDs. Graeve, McGovern, Arnold, et al. (2017) reported that interpersonal influence was significantly associated with PPE use after implementing a quality improvement intervention. The participants of Abu Sharour et al. (2021) and Srisintorn et al. (2021) had moderate interpersonal influence. He et al. (2017) reported a negative interpersonal influence.

### 3.8 Perceived Conflict of Interest

Perceived conflict of interest is defined by Gershon et al. as a conflict “between workers' need to protect themselves and their need to provide medical care to patients” (1995, 225). Khan, Khowaja, and Ali (2012) report that 58% of nurse participants felt that “chemotherapy causes more harm than good,” making them feel guilty. Chen, Lu, and Lee (2016) noted that nurses perceived PPE usage as harming patients psychologically and possibly refusing treatment, specifically with children. The participants believed it was appropriate to avoid using PPE because they were more experienced and always knew how to reduce contamination. Chen, Lu, and Lee (2016) found that pregnancy posed a perceived conflict between a social and professional role in administering chemotherapy and the balance between foetal safety and job protection.

Graeve, McGovern, Alexander, et al. (2017) and Graeve, McGovern, Arnold, et al. (2017) found that perceived conflict of interest was insignificant against all other variables. Callahan et al. (2016) showed that lower conflict of interest was associated with higher knowledge, higher self‐efficacy, low perceived barriers, and better workplace safety. Abu Sharour et al. (2021) reported that conflict of interest negatively predicted safe handling precautions along with perceived risk and age. Srisintorn et al. (2021) showed a small magnitude but statistically significant association with PPE usage. Polovich and Clark (2012) noted that a high conflict of interest was statistically significantly associated with low workplace safety, low interpersonal influences, and low PPE usage.

### 3.9 HD Safe Handling Precautions

Safe handling precautions were the focus of most papers with the exclusion of four studies (Bernabeu‐Martínez et al. 2021; Khan, Khowaja, and Ali 2012; Kutlutürkan and Kirca 2022; and Verity et al. 2008). Recommended safe handling practices were reported as not followed by Abu Sharour et al. (2021), Hon, Teschke, and Shen (2015), He et al. (2017), Kosgeroglu et al. (2006), and Topçu and Beşer (2017).

## 4 Inductive Synthesis

### 4.1 Perceived Solutions

#### 4.1.1 Education and Guidelines

Eleven Studies described that their participants had received formal education training (Alehashem and Baniasadi 2018; DeJoy et al. 2017; Callahan et al. 2016; Constantinidis et al. 2011; Kyprianou et al. 2010; Mahdy, Rahman, and Hassan 2017; Polovich and Clark 2012; Silver, Steege, and Boiano 2016; Simons and Toland 2019; Srisintorn et al. 2021; Verity et al. 2008). Four studies reported that their participant had received in‐service training as education (Alehashem and Baniasadi 2018; Hanafi et al. 2015; Shahrasbi et al. 2014; Tuna and Baykal 2017). Six studies identified that there was a lack of available education (Asefa et al. 2021; Baykal, Seren, and Sokmen 2009; Benoist et al. 2022; Çınar and Karadakovan 2022; Khan, Khowaja, and Ali 2012; Topçu and Beşer 2017). Three studies narrated the education coming from textbooks, internet content, and often unreliable sources (Kyprianou et al. 2010; Shahrasbi et al. 2014; Turk et al. 2004). Graeve, McGovern, Alexander, et al. (2017) and Graeve, McGovern, Arnold, et al. (2017) were the only study to report training and the use of contamination swabbing in clinical areas and pre‐and post‐knowledge tests as an education intervention.

Alehashem and Baniasadi (2018) reported limited association with professional bodies concerning guideline use. DeJoy et al. (2017) reported the most familiarity with the Oncology Nursing Society (ONS) (USA) guidelines, and 81% were familiar with one of the four guidance documents. Three studies reported that guideline knowledge was translated into good practice (Alehashem and Baniasadi 2018; Nwagbo et al. 2017; Silver, Steege, and Boiano 2016). Two studies stated that the information level must be seen in practice (Constantinidis et al. 2011; Kosgeroglu et al. 2006). In the Graeve, McGovern, Alexander, et al. (2017) and Graeve, McGovern, Arnold, et al. (2017) study, despite using ONS recommendations integrated into the study design, high contamination levels were still present, indicating areas for improvement.

#### 4.1.2 Surveillance

Graeve, McGovern, Alexander, et al. (2017) and Graeve, McGovern, Arnold, et al. (2017) reported surveillance as swabbing for environmental contamination to give a targeted intervention to help create awareness amongst the healthcare team in the workplace. In two studies, participants stated that they felt more physiological surveillance should be available (Baykal, Seren, and Sokmen 2009; Constantinidis et al. 2011). In the Chen, Lu, and Lee (2016) study, the participants identified their personal experiences of no side effects following repeated exposure as a justification for their behaviour and overall safety without monitoring.

#### 4.1.3 Hierarchy of Controls Excluding the Use of PPE

The hierarchy of controls has five levels of actions to reduce or remove hazards and lower worker exposure. Based on general effectiveness, the preferred order of action is elimination, substitution, engineering controls, administrative controls, and PPE.

Four studies used biosafety cabinets and isolators to prepare drugs in the preparation phase (Baykal, Seren, and Sokmen 2009; Ben‐Ami et al. 2001; Orujlu et al. 2008; Shahrasbi et al. 2014).

Eight studies indicated the usage of engineering controls to reduce exposure to CDS; Shahrasbi et al. (2014) reported the usage of biosafety cabinets to prepare the CDs, and surface sampling was done to monitor any spillages of the CDs. Baykal et al. (2008), Ben‐Ami et al. (2001), and Orujlu et al. (2008) reported the usage of biosafety cabinets; Chen, Lu, and Lee (2016) reported centralised oncology pharmacy with professional equipment where all the drugs were prepared and sent for administration.

Recent studies by Asefa et al. (2021), DeJoy et al. (2017), Silver, Steege, and Boiano (2016), Simons and Toland (2017, 2019), and Topçu and Beşer (2017) reported the usage of closed system transfer devices (CSTDs) for the preparation and administration of CD.

When referring to the engineering controls of closed system devices and administration of CDs, Asefa et al. (2021) stated that 37 (48.1%) of the respondents used disposable syringes without Luer‐lock fittings during cytotoxic drug administration. DeJoy et al. (2017) reported that 94% of the nurses indicated that they “always” used luer‐lock fittings for needleless systems and 91% claimed that they “always” used needleless systems. Silver, Steege, and Boiano (2016) study found statistically significant reductions in spills when using two types of devices designed to prevent exposure: CTSDs and luer‐lock fittings. Bernabeu‐Martínez et al. (2021) study highlighted that the risk was reduced if associated with a luer‐lock system, with a perception of risk of exposure less for valve systems versus three tree systems.

### 4.2 Side Effects and Risky Behaviours (Inductive)

Sixteen studies reported side effects as health problems due to handling CDs. These adverse effects include weakness, fatigue, sleepiness, loss of hair, headache, nervousness, respiratory problems, nausea, eye irritation, and decreased blood count leading to problems with immunity and anaemia (Batista et al. 2021; Baykal, Seren, and Sokmen 2009; Borges, Silvino, and dos Santos 2015; Constantinidis et al. 2011; Hanafi et al. 2017; He et al. 2017; Kyprianou et al. 2010; Mahdy, Rahman, and Hassan 2017; Orujlu et al. 2016; Shahrasbi et al. 2014; Simons and Toland 2017, 2019; Soheili et al. 2021a; Topçu and Beşer 2017; Tuna and Baykal 2017; Turk et al. 2004). Benoist et al. (2022) reported cutaneous, primarily in burns or tingling sensations, and Tuna and Baykal (2017) reported lip blisters. Kutlutürkan and Kırca (2022) reported psychosocial problems such as burnout syndrome, compassion, and emotional exhaustion. Menstrual cycle irregularities and reproductive issues were reported in seven studies (Borges, Silvino, and dos Santos 2015; Constantinidis et al. 2011; Kyprianou et al. 2010; Mahdy, Rahman, and Hassan 2017; Orujlu et al. 2016; Simons and Toland 2017; Turk et al. 2004). Ben‐Ami et al. (2001) reported that their participants perceived susceptibility increased as much as the body damage potential was tangible and visible, for example, eye splashes, compared to other actions that cannot be monitored or measured.

Three studies reported risky behaviours of nurses in the working areas, such as eating, storing food and beverages, drinking beverages, smoking, and using cosmetics (Baykal, Seren, and Sokmen 2009; Ben‐Ami et al. 2001; Turk et al. 2004). Ben‐Ami et al. (2001) described that older nurses were less likely to perform risky behaviours. They noted a significant correlation between health beliefs and the usage of safety measures, perceived susceptibility, and perceived benefit. They found no connection between the perceived severity of side effects and safe behaviour.

Kosgeroglu et al. (2006) did not find a significant correlation between protection of the environment or self‐associated with experience in the chemotherapy unit, the participant's age, or the education received.

Kosgeroglu et al. (2006) and Ben‐Ami et al. (2001) observed that nurses were more likely to be cautious about preparation rather than administering CD to the patient. Hanafi et al. (2015) attributed the preparation of CDs to the adverse effects suffered by the nurses but were unable to identify the CD responsible.

High contamination levels were observed by Graeve, McGovern, Alexander, et al. (2017) and Graeve, McGovern, Arnold, et al. (2017), indicating risky behaviour in CD checking areas and a lack of double gloving.

## 5 Discussion

This systematic review is the first to utilise a deductive and inductive framework synthesis to understand the perceptions and experiences of cancer nurses of potential occupational exposure when handling CDs worldwide. The deductive synthesis utilising the Theoretical Framework: Factors Predicting the Use of Hazardous Drug (HD) Safe‐Handling Precautions (Figure 3) gave the framework a unique picture of perceived influencing factors, with most studies aimed to explore the outcome of safe handling precautions.

Framework, deductive synthesis revealed 38 global heterogeneous studies from 17 countries with different methodologies, populations, clinical settings, underpinning education and guidance, environmental safety and surveillance practices, and safe handling precautions applied. Like Lin et al. (2019) and Bernabeu‐Martínez et al. (2018), it was deemed challenging to conduct a meta‐analysis. Even the most consistent validated measurement by Polovich and Clark (2012), which was utilised across six studies, revealed the global contextual diversity underscoring the prevalence of complexity arising in this area of research (Abu Sharour et al. 2021; Callahan et al. 2016; Graeve, McGovern, Alexander, et al. 2017; Graeve, McGovern, Arnold, et al. 2017; He et al. 2017; Mahdy, Rahman, and Hassan 2017; and Srisintorn et al. 2021).

This review describes the cancer nursing perception as ‘situational’ inductively. The participants in the 38 studies described individual, shared, and cultural perceptions embodied in different healthcare systems and workplace safety, the use of different clinical guidelines, a variation on the requirement for education, and the ensuing application to their clinical practice. Thirty‐two studies utilised self‐reported, subjective methodology. Therefore, this review raises the point that the responses from cancer nurses are espoused perceptions of what should occur daily.

Supporting this interpretation further are the seven mixed methods (Asefa et al. 2014; Benoist et al. 2022; Chen, Lu, and Lee 2016; Graeve, McGovern, Alexander, et al. 2017; Graeve, McGovern, Arnold, et al. 2017; Polovich and Clark 2012; Soheili et al. 2021a; Topçu and Beşer, 2017) and five observational studies (Chen, Lu, and Lee 2016; Colvin, Karius, and Albert 2016; Hanafi et al. 2017; Kosgeroglu et al. 2006; Shahrasbi et al. 2014) where the attitudes and beliefs and perception of practice changed, when delivering care within a complex environment in different countries. One study by Hanafi et al. (2015) stated that the complexity of the environment resulted in the potential to achieve less than 50% adherence to PPE for preparation and administration. Therefore, it is proposed that perception is espoused because the practice experience differs depending on an individual's daily environmental circumstances.

By being solely reliant on the perceived solutions of education, environmental surveillance, and hierarchy of controls in creating the work safety climate, there is no consideration of the multiple unforeseen clinical tasks requiring priority decision‐making about ‘in‐the‐moment’ safe handling precautions (Fazel et al. 2022), also described as ‘optimising violations’ to get the job done quickly (Reason 1990). Despite the availability of guidelines in most countries (Bernabeu‐Martínez et al. 2018; Coyne et al. 2019; Quispe Condor et al. 2021), in their deliberative process of contextualising policy, literature, and expert opinion, Fazel et al. (2022) uncovered that the most common barriers within the clinical practice were poor training (46%), poor safety culture(41%), and inconsistent policies (36%). All of these factors affect the perception and experience of cancer nurses of potential occupational exposure to CDs. Lin et al. (2017) state three defining characteristics common to the safety climate in healthcare providers: the creation of a safe working environment by senior management (cultural perception) in healthcare organisations, the shared perception of healthcare providers about the safety of their work environment, and the effective dissemination of safety information. In addition, Lin et al. (2017) suggest that organisational influence must provide a positive attitude to improving work safety climate and should monitor environmental equipment and safety management operations. Consideration should be given to the perception of safety climate in the workplace. Compliance with safe work practices and sharing perceptions of work safety with colleagues should serve as a basis for jointly creating a safe working environment.

In this global review, the experience of providing workplace safety and the exploration of working conditions of nurses highlights that cancer nurses are feeling overburdened with the number of patients and workload, resulting in a perceived increase in potential occupational exposure from CDs. The extension of the inclusion criteria in this review generated more nuanced data about working practices that influence the cancer nurse's experience and perception of safe handling practices (Coyne et al. 2019; Lin et al. 2019). The qualitative interview studies indicated that many nurses want a secure environment and better working conditions. Limited, global representative qualitative studies have been conducted from 2015 until 2017 (Chen, Lu, and Lee 2016; Topçu and Beşer 2017; Tuna & Baykal 2017; Verity et al. 2008). There is growing evidence of exploring oncology nurses' broader contextual perceptions regarding occupational needs, work‐related stressors, and health work environment (Arıkan Dönmez et al. 2023; Soheili, Jokar, et al. 2021; Soheili et al. 2021a; Soheili et al. 2021b) and, in addition, the work safety modelling (Lin et al. 2022) and health behaviours determinants scale (Abu‐Alhaija et al. 2022, 2023).

When considering creating a workplace safety solution to enhance the perceptions and experience of cancer nurses, the study by Graeve, McGovern, Alexander, et al. (2017) and Graeve, McGovern, Arnold, et al. (2017) used a two‐armed approach: training and a contamination swabbing exercise. The results were then shared with the administration units to determine a change in practice. The results showed statistical significance in increasing perceived risk on pre and post‐survey questions, resulting in higher use of PPE, but the swab retesting did not support an overall workflow change, with continued contamination. Since 2019, The United States Pharmacopoeia (USP) Chapter < 800 > guidelines (2017) are set to be adopted in the US and Canada, requiring regular surface sampling for antineoplastic drug (AD) surface contamination as a means of environmental surveillance. More contamination studies are being conducted and published, qualifying local variance. Arnold and Kaup (2019) analysis revealed that statistically significant differences were found between cancer nurse chemotherapy clinics in the frequency of contact among nursing staff in patient administration areas for five of the six surfaces. The duration of contact was not significantly different except for the duration of touching the IV pump.

In further support of safe handling practices and potential occupational exposure, Bernabeu‐Martínez et al. (2021) indicate that cancer nurses perceive the specific actions that are out of their control in the process of administration and disposal as riskier. The highest risk is associated with accidental exposure during connection and disconnection of infusion lines, areas around the spike, where there is the risk of exposure by drops and spills, by tares in the infusion bags or inadequate connection. This study only questions the specifics of administration practice and needs to consider the layers of complexity when adding patient and family (human) factors into the process.

This review also spotlights that there may be a counter‐effect to safe handling precautions. Chen, Lu, and Lee's (2016) study shows that experiencing annual surveillance reduces practising safe handling precautions, as the perception is that surveillance will diagnose an individual's occupational exposure to CDs. Furthermore, Topçu and Beşer (2017) identified closed system transfer devices that were perceived as reducing exposure to ‘not possible’, resulting in decreased usage of PPE, with Chen, Lu, and Lee (2016) reporting closed systems ‘encapsulated toxicity’.

Similarly, both Baykal, Seren, and Sokmen (2009) and Turk et al. (2004) reported risky behaviours of nurses in hazardous working areas, such as eating, storing food and beverages, drinking beverages, smoking, and using cosmetics. One interpretation may be that they felt that they were conducting appropriate safe handling precautions, and, therefore, the risky behaviour was not perceived as dangerous but rather a lack of education.

In this review, cancer nurses are perceiving and or experiencing side effects which they attribute to exposure to cytotoxic drugs (Borges, Silvino, and dos Santos 2015; Constantinidis et al. 2011; Hanafi et al. 2017; Kutlutürkan and Kırca 2022; Kyprianou et al. 2010; Mahdy, Rahman, and Hassan 2017; Nwagbo et al. 2017; Orujlu et al. 2016; Shahrasbi et al. 2014; Simons and Toland 2017; Soheili, Jokar, et al. 2021; Soheili et al. 2021a; Soheili et al. 2021b; Topçu and Beşer 2017; Tuna and Baykal 2017; Turk et al. 2004). These are short‐ and longer‐term effects, including reproductive issues and foetal abnormalities. These reported side effects are perceived globally, not confined to any country or workplace. Internationally, it remains challenging to attribute any of the side effects to specific actions or inactions concerning safe handling precautions.

**FIGURE 4:**
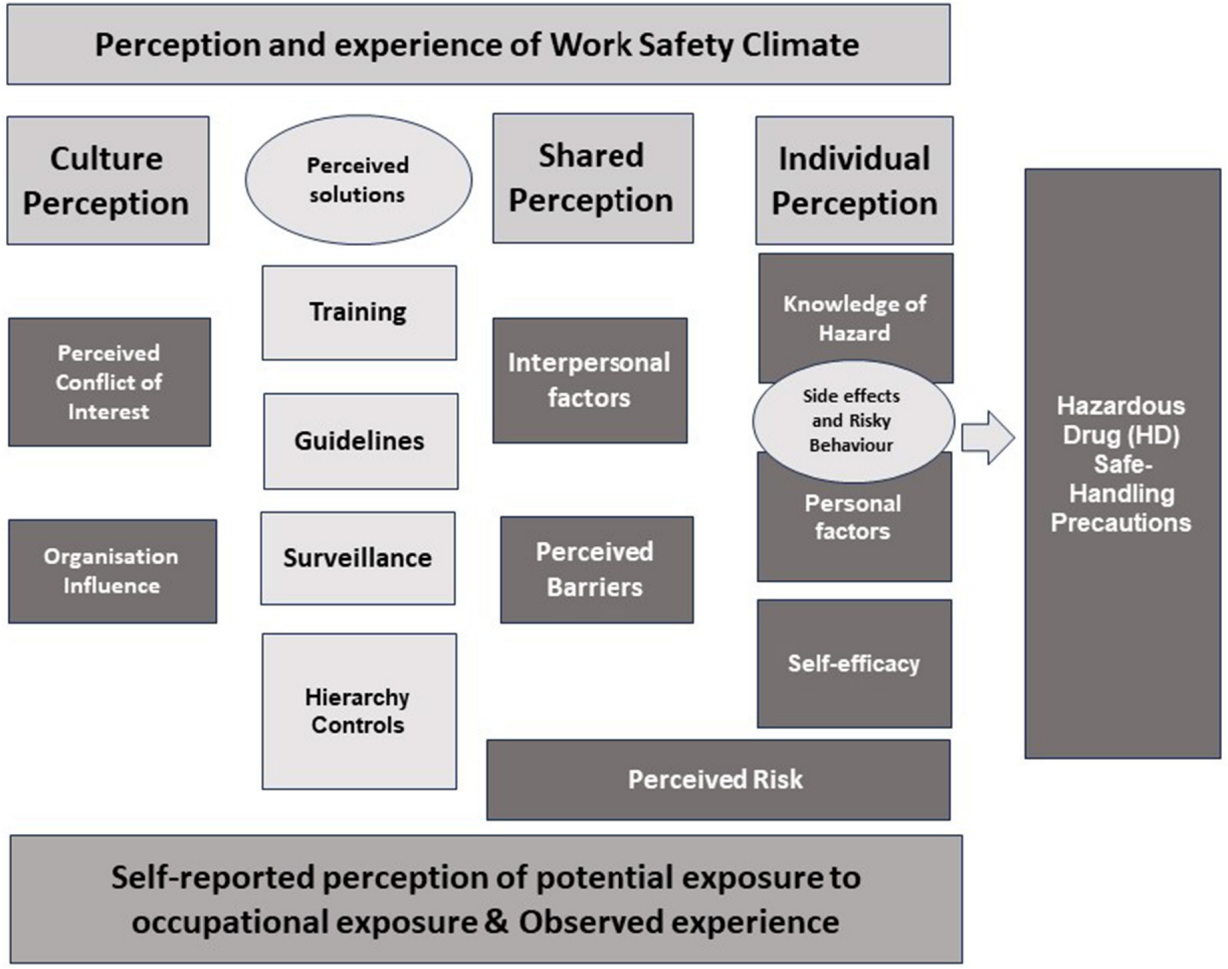
Adapted theoretical framework. [Colour figure can be viewed at wileyonlinelibrary.com]

This review adapted the priori framework (Polovich and Clark 2012) to consider cancer nurses' perceptions and experiences about potential occupational exposure to CDs; the framework represents the concept of the espoused perception of safe practice, which coexists and often conflicts with the experience of conducting safe practice when reducing the potential occupational exposure to CDs (Figure 4).

In this review, the framework was not being tested; we were using it to guide the synthesis of the included studies. The original priori framework (Polovich and Clark 2012) attributes direct links between its elements. This review has detracted from making direct associations between the elements in the model, as this could only be achieved with a robust meta‐analysis. The inductive additions to the framework are lighter in colour than the original priori framework categories.

From left to right (Figure 4), going from espoused perception and experience, working towards hazardous drugs and safe handling precautions is seen at three levels.

The review sees the perception and experience of potential occupational exposure of cancer nurses as a complex intervention and challenge to homogenise within a global context when cancer nursing espoused perception and the expertise in workplace safety and safe handling practice played out differently depending on the country, the unit set, the organisation, and the individual cancer nurses involved. How this complex environment is affected will be determined by the cascade of events and if they result in an occupational exposure event, in short‐term or long‐term exposure for any individual cancer nurse.

### 5.1 Implications for Practice

This review recommends that the theoretical model moves from the safe handling precautions being solely the individual's accountability, where the hierarchy of control is devised, education is delivered, guidance is given, and surveillance is applied, with the outstanding focus being on the ‘why’ individuals are not practising safe handling. This adapted model proposes understanding that individual cancer nurse perception is created from a shared and cultural perception in which the handling CDs is embedded, changing practice in perception and experience of safe handling precautions depending on the workplace safety of the clinical setting and the country.

It is imperative, then, that due to the complexity and differing practices, local units must provide nationally agreed education, guidelines, and appropriate and safe working environments to enable the perception of the correct safety practices, acknowledging that the practical experience is often chaotic and exists in an unpredictable environment. The safe practice has to be monitored against national and international cancer nursing policies and directives. Practice must also be monitored to ensure that knowledge and competency are applied and embedded daily by regularly imposing risk assessment, observation, and simulated activity. Practice alone cannot rely on the perceived solutions of education and guidance, with the responsibility and accountability being on the cancer nurse. Practice experience must be monitored against the cultural and shared perception influencing practice.

Further testing of this theoretical model is necessary to understand the complexity of the working environment and more innovative educational approaches to embed safety practices in it. Furthermore, future research should focus on quality improvement contamination swapping activity and more inexpensive and immediate innovations to detect occupational exposure to cytotoxic drugs.

## 6 Conclusions

Occupational cytotoxic exposure is a reality globally. We have gained new insights on this topic by conducting this framework synthesis review to understand cancer nurses' experiences and perceptions of potential occupational exposure to cytotoxic drugs worldwide. This review reflects the heterogeneous practice and how this is measured about safe handling precautions, including the diversity in perception and experience in knowledge, perceived barriers, perceived risk, self‐efficacy, organisational influence and interpersonal influence, and perceived conflict globally. The review identified further categories of education, guidance, surveillance, hierarchy of controls, risky behaviour, and side effects. This review continues to prove that there is a challenge to standardised international improvement and urges practice to guide safety and well‐being when administering cytotoxic drugs, locally and nationally.

### 6.1 Limitations

Using framework analysis benefited the review by providing a deductive and inductive approach, giving meaning relevant to the research topic under investigation. However, the framework approach is based upon one framework, and the resulting adaption must still be tested in practice. Limitations were found in the nature of the studies as this was dominated by self‐reported data, which again needs to be more generalisable in practice but gives a key indication for practice. The topic of perception and experience is subjective, and the findings would not be generalisable globally but would need to be repeated and interpreted locally. Another limitation was that all non‐English‐written papers were excluded from the review, which would have been applicable when reviewing the abstract. This limitation was most evident from the Asian research studies.

## Author Contributions

K.C. lead author and design, development of protocol, review of articles for inclusion, data extraction, synthesis and write up. J.A. review of protocol and articles for inclusion. M.D. review of articles for inclusion, quality assurance, editing. M.K. designed search strategy and generated articles for review. D.D. review of articles for inclusion, data extraction, synthesis and write up.

## Conflicts of Interest

The authors declare no conflicts of interest.

## Supporting information


Appendix S1

Appendix S2

Appendix S3

## Data Availability

The data that support the findings of this study are available from the corresponding author upon reasonable request.

## References

[jocn17488-bib-0001] Abu Sharour, L. , M. Subih , A. Bani Salameh , and M. Malak . 2021. “Predictors of Chemotherapy Safe‐Handling Precautions and Knowledge Among a Sample of Jordanian Oncology Nurses: A Model‐Building Approach.” Workplace Health & Safety 69, no. 3: 115–123. 33446086 10.1177/2165079920959991

[jocn17488-bib-0002] Abu‐Alhaija, D. , T. Bakas , E. Shaughnessy , and E. Miller . 2023. “The Factors That Influence Chemotherapy Exposure Among Nurses: An Integrative Review.” Workplace Health & Safety 71, no. 5: 212–227. 10.1177/21650799221140583. 36703295 PMC10834144

[jocn17488-bib-0003] Abu‐Alhaija, D. , E. Miller , T. Bakas , and E. Shaughnessy . 2022. “The Development and the Content Validation of the Oncology Nurses Health Behaviors Determinants Scale.” Seminars in Oncology Nursing 38, no. 6: 2022. 10.1016/j.soncn.2022.151317. PMC10823865 35871026

[jocn17488-bib-0004] Alehashem, M. , and S. Baniasadi . 2018. “Safe Handling of Antineoplastic Drugs in the University Hospitals: A Descriptive Survey Study Among Oncology Nurses.” International Journal of Cancer Management 11, no. 2: e6482. 10.5812/ijcm.6482.

[jocn17488-bib-0005] Arıkan Dönmez, A. , Ö. Ovayolu , N. Ovayolu , et al. 2023. “Quality of Work Life and Working Conditions Among Oncology Nurses: A National Online Descriptive Cross‐Sectional Study.” Archives of Environmental & Occupational Health 78, no. 3: 131–141. 10.1080/19338244.2022.2063240. 35412450

[jocn17488-bib-0006] Arnold, S. , and H. Kaup . 2019. “Assessing Variability of Antineoplastic Drugs Handling Practices in Clinical Settings.” Journal of Occupational and Environmental Hygiene 16, no. 12: 757–762. 10.1080/15459624.2019.1667502. 31621520

[jocn17488-bib-0007] Asefa, S. , F. Aga , N. G. Dinegde , and T. G. Demie . 2021. “Knowledge and Practices on the Safe Handling of Cytotoxic Drugs Among Oncology Nurses Working at Tertiary Teaching Hospitals in Addis Ababa, Ethiopia.” Drug, Healthcare, and Patient Safety 13: 71–80. 33833583 10.2147/DHPS.S289025 PMC8019613

[jocn17488-bib-0008] Batista, K. C. , K. H. J. F. Sousa , C. A. D. S. Ruas , and R. C. G. Zeitoune . 2022. “Knowledge About Antineoplastic Drugs: Implications for the Health of Nursing Workers in a General Hospital.” Revista Brasileira de Enfermagem 75, no. 3: e20210025. 10.1590/0034-7167-2021-0025 34669829

[jocn17488-bib-0009] Baykal, U. , S. Seren , and S. Sokmen . 2009. “A Description of Oncology Nurses' Working Conditions in Turkey.” European Journal of Oncology Nursing 13, no. 5: 368–375. 19520605 10.1016/j.ejon.2009.04.004

[jocn17488-bib-0010] Ben‐Ami, S. , J. Shaham , S. Rabin , A. Melzer , and J. Ribak . 2001. “The Influence of Nurses' Knowledge, Attitudes, and Health Beliefs on Their Safe Behavior With Cytotoxic Drugs in Israel.” Cancer Nursing 24, no. 3: 192–200. 11409063

[jocn17488-bib-0011] Benoist, H. , A. Busson , A. Faveyrial , et al. 2022. “Perception, Knowledge, and Handling Practice Regarding the Risk of Exposure to Antineoplastic Drugs in Oncology Day Hospitalisation Units and Compounding Unit Staff.” Journal of Oncology Pharmacy Practice. 10.1177/10781552221103803. 35635230

[jocn17488-bib-0012] Bernabeu‐Martínez, M. A. , M. Ramos Merino , J. M. Santos Gago , L. M. Álvarez Sabucedo , C. Wanden‐Berghe , and J. Sanz‐Valero . 2018. “Guidelines for Safe Handling of Hazardous Drugs: A Systematic Review.” PLoS One 13, no. 5: e0197172. 10.1371/journal.pone.0197172. 29750798 PMC5947890

[jocn17488-bib-0013] Bernabeu‐Martínez, M. Á. , J. Sánchez‐Tormo , P. García‐Salom , J. Sanz‐Valero , and C. Wanden‐Berghe . 2021. “Perception of Risk of Exposure in the Management of Hazardous Drugs in Home Hospitalisation and Hospital Units.” PLoS One 16, no. 7: e0253909. 10.1371/journal.pone.0253909. 34197532 PMC8248625

[jocn17488-bib-0014] Borges, G. G. , Z. R. Silvino , and L. C. G. dos Santos . 2015. “Proposal for Best Practice Guidelines on Chemical Exposure Risk for Nurses of a Chemotherapy Unit.” Revista De Pesquisa Cuidado é Fundamental Online 7, no. 4: 3506–3515. 10.9789/2175-5361.2015.v7i4.3506-3515.

[jocn17488-bib-0015] Callahan, A. , N. Ames , M. L. Manning , K. Touchton‐leonard , L. Yang , and G. R. Wallen . 2016. “Factors Influencing Nurses' Use of Hazardous Drug Safe‐Handling Precautions.” Oncology Nursing Forum 43, no. 3: 342–349. 10.1188/16.ONF.43-03AP. 27105195 PMC4876597

[jocn17488-bib-0016] Carroll, C. , A. Booth , J. Leaviss , and J. Rick . 2013. “"Best Fit" Framework Synthesis: Refining the Method.” BMC Medical Research Methodology 13: 37. 10.1186/1471-2288-13-37. 23497061 PMC3618126

[jocn17488-bib-0017] Chen, H. C. , Z. Y. Lu , and S. H. Lee . 2016. “Nurses' Experiences in Safe Handling of Chemotherapeutic Agents: The Taiwan Case.” Cancer Nursing 39, no. 5: E29–E38. 10.1097/NCC.0000000000000314. 26496520

[jocn17488-bib-0018] Çınar, D. , and A. Karadakovan . 2022. “Investigation of Occupational Safety in Oncology Nurses.” International Journal of Occupational Safety and Ergonomics: JOSE 28, no. 3: 1750–1755. 10.1080/10803548.2021.1928405. 33970804

[jocn17488-bib-0019] Colvin, C. M. , D. Karius , and N. M. Albert . 2016. “Nurse Adherence to Safe‐Handling Practices: Observation Versus Self‐Assessment.” Clinical Journal of Oncology Nursing 20, no. 6: 617–622. 10.1188/16.CJON.617-622. 27857252

[jocn17488-bib-0020] Connor, T. H. , and M. A. McDiarmid . 2006. “Preventing Occupational Exposures to Antineoplastic Drugs in Health Care Settings.” CA: A Cancer Journal for Clinicians 56, no. 6: 354–365. 17135692 10.3322/canjclin.56.6.354

[jocn17488-bib-0021] Constantinidis, T. C. , E. Vagka , P. Dallidou , et al. 2011. “Occupational Health and Safety of Personnel Handling Chemotherapeutic Agents in Greek Hospitals.” European Journal of Cancer Care 20, no. 1: 123–131. 20148939 10.1111/j.1365-2354.2009.01150.x

[jocn17488-bib-0022] Control of Substances Hazardous to Health Regulations (COSHH) . 2002. https://www.hse.gov.uk/coshh/.

[jocn17488-bib-0023] Coyne, E. , S. Northfield , K. Ash , and L. Brown‐West . 2019. “Current Evidence of Education and Safety Requirements for the Nursing Administration of Chemotherapy: An Integrative Review.” European Journal of Oncology Nursing 41: 24–32. 10.1016/j.ejon.2019.05.001. 31358254

[jocn17488-bib-0024] DeJoy, D. M. , T. D. Smith , H. Woldu , M. A. Dyal , A. L. Steege , and J. M. Boiano . 2017. “Effects of Organisational Safety Practices and Perceived Safety Climate on PPE Usage, Engineering Controls, and Adverse Events Involving Liquid Antineoplastic Drugs Among Nurses.” Journal of Occupational and Environmental Hygiene 14, no. 7: 485–493. 10.1080/15459624.2017.1285496. 28326998

[jocn17488-bib-0025] Eisenberg, S. 2016. “A Call to Action for Hazardous Drug Safety: Where we Have Been and Where we Are Now.” Clinical Journal of Oncology Nursing 20, no. 4: 20–4AP. 10.1188/16.CJON.20-04AP. 27441503

[jocn17488-bib-0026] Eisenberg, S. , and C. Klein . 2021. “Safe Handling of Hazardous Drugs in Home Infusion.” Journal of Infusion Nursing 44, no. 3: 137–146. 10.1097/NAN.0000000000000424. 33935248

[jocn17488-bib-0027] Fazel, S. S. , A. Keefe , A. Shareef , et al. 2022. “Barriers and Facilitators for the Safe Handling of Antineoplastic Drugs.” Journal of Oncology Pharmacy Practice 28, no. 8: 1709–1721. 10.1177/10781552211040176. 34612752

[jocn17488-bib-0028] Fereday, J. , and E. Muir‐Cochrane . 2006. “Demonstrating Rigor Using Thematic Analysis: A Hybrid Approach of Inductive and Deductive Coding and Theme Development.” International Journal of Qualitative Methods 5, no. 1: 80–92. 10.1177/160940690600500107.

[jocn17488-bib-0029] Field, A. , G. Hughes , and S. Rowland . 2017. “A Strategy for Formulating Regulation on CSTDs.” British Journal of Nursing 26, no. Suppl 16b: S15–S22. 10.12968/bjon.2017.26.Sup16b. 28981323

[jocn17488-bib-0030] Gale, N. K. , G. Heath , E. Cameron , S. Rashid , and S. Redwood . 2013. “Using the Framework Method for the Analysis of Qualitative Data in Multi‐Disciplinary Health Research.” BMC Medical Research Methodology 13: 117. 10.1186/1471-2288-13-117. 24047204 PMC3848812

[jocn17488-bib-0031] Gershon, R. R. , D. Vlahov , S. A. Felknor , et al. 1995. “Compliance With Universal Precautions Among Health Care Workers at Three Regional Hospitals.” American Journal of Infection Control 23: 225–236. 7503434 10.1016/0196-6553(95)90067-5

[jocn17488-bib-0032] Graeve, C. , P. M. McGovern , S. Arnold , and M. Polovich . 2017. “Testing an Intervention to Decrease Healthcare Workers' Exposure to Antineoplastic Agents.” Oncology Nursing Forum 44, no. 1: E10–E19. 10.1188/17.ONF.E10-E19. 27991608

[jocn17488-bib-0033] Graeve, C. U. , P. M. McGovern , B. Alexander , T. Church , A. Ryan , and M. Polovich . 2017. “Occupational Exposure to Antineoplastic Agents.” Workplace Health & Safety 65, no. 1: 9–20. 10.1177/2165079916662660. 27758934

[jocn17488-bib-0034] Granikov, V. , R. El Sherif , F. Bouthillier , and P. Pluye . 2022. “Factors and Outcomes of Collaborative Information Seeking: A Mixed Studies Review With a Framework Synthesis.” Journal of Association of Information Science and Technology 73: 542–560. 10.1002/asi.24596.

[jocn17488-bib-0035] Gurusamy, K. S. , L. M. Best , C. Tanguay , E. Lennan , M. Korva , and J. F. Bussières . 2018. “Closed‐System Drug‐Transfer Devices Plus Safe Handling of Hazardous Drugs Versus Safe Handling Alone for Reducing Exposure to Infusional Hazardous Drugs in Healthcare Staff.” Cochrane Database of Systematic Reviews 3, no. 3: CD012860. 10.1002/14651858.CD012860.pub2. 29582940 PMC6360647

[jocn17488-bib-0036] Hanafi, S. , H. Torkamandi , S. Bagheri , M. Tavakoli , N. Hadavand , and M. Javadi . 2015. “Safe Handling of Cytotoxic Drugs and Risk of Occupational Exposure to Nursing Staffs.” Journal of Pharmaceutical Care 3, no. 1–2: 11–15.

[jocn17488-bib-0037] Hawker, S. , S. Payne , C. Kerr , M. Hardey , and J. Powell . 2002. “Appraising the Evidence: Reviewing Disparate Data Systematically.” Qualitative Health Research 12, no. 9: 1284–1299. 10.1177/1049732302238251. 12448672

[jocn17488-bib-0038] He, B. , K. Mendelsohn‐Victor , M. C. McCullagh , and C. R. Friese . 2017. “Personal Protective Equipment Use and Hazardous Drug Spills Among Ambulatory Oncology Nurses.” Oncology Nursing Forum 44, no. 1: 60–65. 10.1188/17.ONF.60-65. 28067030 PMC5225785

[jocn17488-bib-0039] Health Improvement Scotland . 2019. “Closed System Transfer‐Devices for Limiting Exposure to Cytotoxic Anticancer Drugs in Healthcare Professionals, Patients, and Visitors.” Evidence Synthesis. https://cytoprevent.eu/wp‐content/uploads/2021/02/Closed‐system‐transfer‐devices‐for‐limiting‐exposure‐Evidence‐Synthesis‐06‐19‐Scotland.pdf.

[jocn17488-bib-0040] Hon, C. Y. , K. Teschke , and H. Shen . 2015. “Health Care Workers' Knowledge, Perceptions, and Behaviors Regarding Antineoplastic Drugs: Survey From British Columbia, Canada.” Journal of Occupational and Environmental Hygiene 12, no. 10: 669–677. 10.1080/15459624.2015.1029618. 25897641

[jocn17488-bib-0041] Hu, J. , F. Zhao , L. Liu , H. Huang , and X. Huang . 2023. “The Meta‐Analysis of Sister Chromatid Exchange as a Biomarker in Healthcare Workers With Occupational Exposure to Antineoplastic Drugs.” Medicine 102, no. 34: e34781. 10.1097/MD.0000000000034781. 37653817 PMC10470682

[jocn17488-bib-0042] Khan, N. , K. Z. Khowaja , and T. S. Ali . 2012. “Assessment of Knowledge, Skill, and Attitude of Oncology Nurses in Chemotherapy Administration in Tertiary Hospital Pakistan.” Open Journal of Nursing 2, no. 2: 97–103. https://ecommons.aku.edu/pakistan_fhs_son/159.

[jocn17488-bib-0043] Kim, O. , H. Lee , H. Jung , H. J. Jang , Y. Pang , and H. Cheong . 2019. “Korean Nurses' Adherence to Safety Guidelines for Chemotherapy Administration.” European Journal of Oncology Nursing 40: 98–103. 10.1016/j.ejon.2019.04.002. 31229212

[jocn17488-bib-0044] Kosgeroglu, N. , U. Ayranci , N. Ozerdogan , and C. Demirustu . 2006. “Turkish Nurses' Information About and Administration of Chemotherapeutic Drugs.” Journal of Clinical Nursing 15, no. 9: 1179–1187. 10.1111/j.1365-2702.2006.01305.x. 16911059

[jocn17488-bib-0045] Kutlutürkan, S. , and K. Kırca . 2022. “Strengths, Weaknesses, Opportunities, and Threats Analysis of Being an Oncology Nurse: A Turkish Oncology Nurses' Perspective.” International Journal of Palliative Nursing 28, no. 5: 222–231. 10.12968/ijpn.2022.28.5.222. 35648678

[jocn17488-bib-0046] Kyprianou, M. , M. Kapsou , V. Raftopoulos , and E. S. Soteriades . 2010. “Knowledge, Attitudes, and Beliefs of Cypriot Nurses on the Handling of Antineoplastic Agents.” European Journal of Oncology Nursing 14, no. 4: 278–282. 10.1016/j.ejon.2010.01.025. 20299284

[jocn17488-bib-0047] Lester, J. 2012. “Safe Handling and Administration Considerations of Oral Anticancer Agents in the Clinical and Home Setting.” Clinical Journal of Oncology Nursing 16, no. 6: E192–E197. 10.1188/12.CJON.E192-E197. 23178361

[jocn17488-bib-0048] Lin, Y. , Y. Chang , Y. C. Lin , and M. F. Lou . 2019. “Factors Influencing Nurses' Use of Hazardous Drug Safe Handling Precautions.” Oncology Nursing Forum 46, no. 3: E86–E97. 10.1188/19.ONF.E86-E97. 31007261

[jocn17488-bib-0049] Lin, Y. S. , B. S. Gau , H. C. Chen , et al. 2022. “The Relationship Between Safety Climate and Nurses' Safe Handling of Chemotherapy: A Partial Least Squares Structural Equation Modelling Analysis.” European Journal of Oncology Nursing 61: 102222. 10.1016/j.ejon.2022.102222. 36223659

[jocn17488-bib-0050] Lin, Y. S. , Y. C. Lin , and M. F. Lou . 2017. “Concept Analysis of Safety Climate in Healthcare Providers.” Journal of Clinical Nursing 26, no. 11–12: 1737–1747. 10.1111/jocn.13641. 27862495

[jocn17488-bib-0051] Mahdy, N. E. , A. A. Rahman , and H. A. Hassan . 2017. “Cytotoxic Drugs Safety Guidelines: Its Effect on Awareness and Safe Handling Practices of Oncology Nurses.” IOSR Journal of Nursing and Health Science 6: 22–33.

[jocn17488-bib-0052] Mathias, P. I. , B. A. MacKenzie , C. A. Toennis , and T. H. Connor . 2019. “Survey of Guidelines and Current Practices for Safely Handling Antineoplastic and Other Hazardous Drugs Used in 24 Countries.” Journal of Oncology Pharmacy Practice 25, no. 1: 148–162. 10.1177/1078155217726. 28841099 PMC7895469

[jocn17488-bib-0053] McDiarmid, M. A. , M. S. Oliver , T. S. Roth , B. Rogers , and C. Escalante . 2010. “Chromosome 5 and 7 Abnormalities in Oncology Personnel Handling Anticancer Drugs.” Journal of Occupational and Environmental Medicine 52, no. 10: 1028–1034. 10.1097/JOM.0b013e3181f73ae6. 20881619

[jocn17488-bib-0054] Meade, E. 2014. “Avoiding Accidental Exposure to Intravenous Cytotoxic Drugs.” British Journal of Nursing 23, no. 16: S34–S39. 10.12968/bjon.2014.23.Sup16.S34. 25203853

[jocn17488-bib-0055] Meade, E. , A. Simons , and S. Toland . 2017. “The Need for National Mandatory Guidance on CSTDs.” British Journal of Nursing 26, no. Suppl 16b: S5–S14. 10.12968/bjon.2017.26.Sup16b.S5. 28981322

[jocn17488-bib-0057] NIOSH . 2004. “Alert: Preventing Occupational Exposures to Antineoplastic and Other Hazardous Drugs in Health Care Settings.” NIOSH, Pub, 165. https://www.cdc.gov/NIOSH/DOCS/2004‐165/pdfs/2004‐165sum.pdf.

[jocn17488-bib-0058] Nwagbo, S. E. , R. E. Ilesanmi , B. M. Ohaeri , and A. O. Oluwatosin . 2017. “Knowledge of Chemotherapy and Occupational Safety Measures Among Nurses in Oncology Units.” Journal of Clinical Sciences 14, no. 3: 131.

[jocn17488-bib-0059] Oncology Nursing Society . 2019. “Ensuring Healthcare Worker Safety When Handling Hazardous Drugs.” Oncology Nursing Forum 46, no. 6: 647–648. 10.1188/19.ONF.647-648. 31626619

[jocn17488-bib-0060] Orujlu, S. , H. Habibzadeh , M. J. Z. Sakhvidi , and M. Hajaghazadeh . 2016. “Knowledge, Attitude, and Performance of Oncology Nurses Handling Antineoplastic Drugs in Urmia University, Iran Hospitals.” International Journal of Occupational Hygiene 8, no. 1: 14–21.

[jocn17488-bib-0061] Page, M. J. , J. E. McKenzie , P. M. Bossuyt , et al. 2021. “The PRISMA 2020 Statement: An Updated Guideline for Reporting Systematic Reviews.” BMJ 372: n71. 10.1136/bmj.n71. 33782057 PMC8005924

[jocn17488-bib-0062] Polovich, M. 2004. “Safe Handling of Hazardous Drugs.” Online Journal of Issues in Nursing 9, no. 3: 6. 10.3912/OJIN.Vol9No03Man05. 15482092

[jocn17488-bib-0063] Polovich, M. , and P. C. Clark . 2012. “Factors Influencing Oncology Nurses' Use of Hazardous Drug Safe‐Handling Precautions.” Oncology Nursing Forum 39, no. 3: E299–E309. 10.1188/12.ONF.E299-E309. 22543401

[jocn17488-bib-0065] Quispe Condor, Y. S. , L. E. García Saavedra , J. E. Rodríguez Zambrano , M. B. Espinoza Acuña , and O. G. Bedoya Ticlavilca . 2021. “Standards for the Safe Administration of Chemotherapy in Oncological Patients 2015–2020: A Systematic Review.” Journal of Global Health and Medicine 5, no. 2: 50–65. 10.32829/ghmj.v5i2.

[jocn17488-bib-0066] Reason, J. 1990. “The Contribution of Latent Human Failures to the Breakdown of Complex Systems.” Philosophical Transactions of the Royal Society of London. Series B, Biological Sciences 327: 475–484. 10.1098/rstb.1990.0090. 1970893

[jocn17488-bib-0067] Rudnitzki, T. , and D. McMahon . 2015. “Oral Agents for Cancer: Safety Challenges and Recommendations.” Clinical Journal of Oncology Nursing 19, no. 3 Suppl: 41–46. 10.1188/15.S1.CJON.41-46. 26030392

[jocn17488-bib-0068] Shahrasbi, A. A. , M. Afshar , F. Shokraneh , et al. 2014. “Risks to Health Professionals From Hazardous Drugs in Iran: A Pilot Study of Understanding of Healthcare Team to Occupational Exposure to Cytotoxics.” EXCLI Journal 13: 491–501. 26417276 PMC4464082

[jocn17488-bib-0069] Silver, S. R. , A. L. Steege , and J. M. Boiano . 2016. “Predictors of Adherence to Safe Handling Practices for Antineoplastic Drugs: A Survey of Hospital Nurses.” Journal of Occupational and Environmental Hygiene 13, no. 3: 203–212. 10.1080/15459624.2015.1091963. 26556549

[jocn17488-bib-0070] Simons, A. , and S. Toland . 2017. “Perceived Adverse Effects From Handling Systemic Anticancer Therapy Agents.” British Journal of Nursing (Mark Allen Publishing) 26, no. 16: S38–S44. 10.12968/bjon.2017.26.16.S38. 28880622

[jocn17488-bib-0071] Simons, A. , and S. Toland . 2019. “Nurses' Safety During Cancer Therapy Must Be a Priority.” Cancer Nursing Practice 18, no. 1: 14. 10.7748/cnp.18.1.14.s14.

[jocn17488-bib-0072] Soheili, M. , F. Jokar , M. Eghbali‐Babadi , M. Sharifi , and F. Taleghani . 2021. “Exploring the Occupational Health Needs of Oncology Nurses: A Qualitative Study.” Journal of Education and Health Promotion 10: 224. 10.4103/jehp.jehp_1151_20. 34395661 PMC8318182

[jocn17488-bib-0073] Soheili, M. , F. Taleghani , F. Jokar , M. Eghbali‐Babadi , and M. Sharifi . 2021a. “Occupational Stressors in Oncology Nurses: A Qualitative Descriptive Study.” Journal of Clinical Nursing 30, no. 21–22: 3171–3181. 10.1111/jocn.15816. 33960034

[jocn17488-bib-0074] Soheili, M. , F. Taleghani , F. Jokar , M. Eghbali‐Babadi , and M. Sharifi . 2021b. “Oncology Nurses' Needs Respecting Healthy Work Environment in Iran: A Descriptive Exploratory Study.” Asia‐Pacific Journal of Oncology Nursing 8, no. 2: 188–196. 10.4103/apjon.apjon_64_20. 33688568 PMC7934596

[jocn17488-bib-0075] Srisintorn, W. , A. Geater , M. Polovich , and P. Thongsuksai . 2021. “Factors Influencing Precautions Against Antineoplastic Drug Exposure Among Nurses and Nurse Assistants in Thailand.” International Archives of Occupational and Environmental Health 94, no. 5: 813–822. 10.1007/s00420-020-01649-9. 33427994

[jocn17488-bib-0076] Topçu, S. , and A. Beşer . 2017. “Oncology Nurses' Perspectives on Safe Handling Precautions: A Qualitative Study.” Contemporary Nurse 53, no. 3: 271–283. 10.1080/10376178.2017.1315828. 28387169

[jocn17488-bib-0077] Tuna, R. , and U. Baykal . 2017. “A Qualitative Study: Determination of the Working Conditions and Knowledge Levels of Oncology Nurses in Terms of Employee Safety.” International Journal of Nursing Clinical Practices 4: 231. 10.15344/2394-4978/2017/231.

[jocn17488-bib-0078] Turk, M. , A. Davas , M. Ciceklioglu , F. Sacaklioglu , and T. Mercan . 2004. “Knowledge, Attitude, and Safe Behaviour of Nurses Handling Cytotoxic Anticancer Drugs in Ege University Hospital.” Asian Pacific Journal of Cancer Prevention: APJCP 5, no. 2: 164–168. 15244519

[jocn17488-bib-0079] United States Convention Pharmacopeia USP General Chapter <800> . 2017. “Handlining in Health Care Setting.” https://www.usp.org/compounding/general‐chapter‐hazardous‐drugs‐handling‐healthcare.

[jocn17488-bib-0080] Verity, R. , T. Wiseman , E. Ream , E. Teasdale , and A. Richardson . 2008. “Exploring the Work of Nurses Who Administer Chemotherapy.” European Journal of Oncology Nursing 12, no. 3: 244–252. 10.1016/j.ejon.2008.02.001. 18467173

[jocn17488-bib-0081] Yu, E. 2020. “Occupational Exposure in Health Care Personnel to Antineoplastic Drugs and Initiation of Safe Handling in Hong Kong: A Literature Review.” Journal of Infusion Nursing 43, no. 3: 121–133. 10.1097/NAN.0000000000000361. 32287167

