# Supplementary material for: Global Cancer Nurse's Experiences and Perceptions of Potential Occupational Exposure to Cytotoxic Drugs: Mixed Method Systematic Review With Framework Synthesis
Source: J Clin Nurs. 2024 Oct 29;33(12):4585–601. doi: 10.1111/jocn.17488 (PMC11579580; doi:10.1111/jocn.17488)
Supplement: Supplementary file 1 — Appendix S1 [file JOCN-33-4585-s002.docx]

# Supplementary material: Search Strategy

CINAHL with Full text (EBSCO), PubMed (including Medline and PMC), Web of Science, Ovid Nursing, PsycINFO.

Journal hand searching will also be done in key cancer and oncology journals that are not indexed in any of the above databases, as well as forwards and backwards citation searching.

Grey literature searching will also be undertaken to identify key research reports by relevant professional organisations e.g. European Oncology Nursing Society, Oncology Nursing Society. Any other grey literature sources in primary research format will be considered for inclusion, including theses, conference papers, and pre-prints.

Sources will be limited to those published in English and those published from 2001 onwards.

17. Search Strategy – keyword concepts

| Main Concept | Cancer Nurses | Occupational Exposure | Cytotoxic drugs | Experiences |
| --- | --- | --- | --- | --- |
| Subject Headings | Oncologic nursing (CINAHL)  Oncology Nursing (Medline) | Occupational exposure | Cytotoxins  Carcinogens  Teratogens  Antineoplastic agents (explode) | Perceptions  Views  Opinions  Thoughts  Perspectives  Feelings  Attitudes  Knowledge |
| Free Text Search Terms | Oncology nurs*  Cancer nurs* | Workplace exposure  Potential exposure  Occupational Hazard  Occupational Risk  Staff exposure  Nurse safety  Safe handling | Chemotherapy drugs  Toxic drugs  Chemotherapy Medication  Antineoplastic drugs  Antineoplastics  Antineoplastic Agents  Anticancer drugs  Hazardous drugs  Carcinogens  Genotoxicity  Teratogenicity  Teratogens  cytotoxins |  |

Search undertaken 11^th^ Feb 2022

| Cancer nursing | 1 | (MH "Oncologic Nursing+") | 17,465 |
| --- | --- | --- | --- |
|  | 2 | TI oncolog* N2 nurs* OR AB oncolog* N2 nurs* | 7,766 |
|  | 3 | TI cancer N2 nurs* OR AB cancer N2 nurs* | 4,284 |
|  | 4 | S1 OR S2 OR S3 | 22,364 |
| Occupational exposure | 5 | (MH "Occupational Exposure") | 20,300 |
|  | 6 | TI work* N2 exposure OR AB work* N2 exposure | 3,470 |
|  | 7 | TI potential exposure OR AB potential exposure | 3,795 |
|  | 8 | TI occupation* N2 hazard* OR AB occupation* N2 hazard* | 1,471 |
|  | 9 | TI occupation* N2 risk* OR AB occupation* N2 risk* | 2,913 |
|  | 10 | TI staff* N2 exposure OR AB staff* N2 exposure | 310 |
|  | 11 | TI nurs* N2 safety OR AB nurs* N2 safety | 2,670 |
|  | 12 | TI safe handling OR AB safe handling | 1,232 |
|  | 13 | S5 OR S6 OR S7 OR S8 OR S9 OR S10 OR S11 OR S12 | 31,857 |
| Cytotoxic drugs | 14 | (MH "Cytotoxins") OR (MH "Carcinogens") OR (MH "Teratogens") OR (MH "Antineoplastic Agents+") | 133,934 |
|  | 15 | TI chemotherapy drugs or toxic drugs or chemotherapy medication or antineoplastic drugs or antineoplastic agents or antineoplastics or anticancer drugs or hazardous drugs or carcinogens or genotoxicity or teratogenicity or teratogens or cytotoxic drugs or cytotoxins | 2,680 |
|  | 16 | AB chemotherapy drugs or toxic drugs or chemotherapy medication or antineoplastic drugs or antineoplastic agents or antineoplastics or anticancer drugs or hazardous drugs or carcinogens or genotoxicity or teratogenicity or teratogens or cytotoxic drugs or cytotoxins | 11,084 |
|  | 17 | S14 OR S15 OR S16 | 140,737 |
| Experiences & Perception | 18 | TI experiences or Perceptions or Views or Opinions or Thoughts or Perspectives or Feelings or Attitudes or Knowledge | 323,774 |
|  | 19 | AB experiences or Perceptions or Views or Opinions or Thoughts or Perspectives or Feelings or Attitudes or Knowledge | 806,683 |
|  | 20 | S18 or S19 | 969,835 |
| All terms combined | 21 | S4 AND S13 AND S17 AND S20 | 48 |
| Language Limited to English | 22 | S4 AND S13 AND S17 AND S20 | 42 |
| Date from 2000 | 23 | S4 AND S13 AND S17 AND S20 | 41 |
