# Supplementary material for: Global Cancer Nurse's Experiences and Perceptions of Potential Occupational Exposure to Cytotoxic Drugs: Mixed Method Systematic Review With Framework Synthesis
Source: J Clin Nurs. 2024 Oct 29;33(12):4585–601. doi: 10.1111/jocn.17488 (PMC11579580; doi:10.1111/jocn.17488)
Supplement: Supplementary file 3 — Appendix S3 [file JOCN-33-4585-s001.docx]

| **Name of the Study** | | **Sample of Nurses** | | **Aim** | | **Characteristics under the study** | | **Methodology used** | | **Theoretical model** | | **Research setting** | **Tools** | | **Hawker score (Max 27)** | |  |
| --- | --- | --- | --- | --- | --- | --- | --- | --- | --- | --- | --- | --- | --- | --- | --- | --- | --- |
| **Abu-Sharour et al. (2021) Jordan** | | | 153 | | To examine knowledge of safe-handling precautions among a sample of oncology nurses | | Knowledge, safe handling precautions | | Quantitative Cross-sectional study | | Factors Predicting Use of Hazardous Drug (HD) Safe-Handling Precautions | | Nurses who were employed in two governmental hospitals in  Jordan. | Factors Predicting Use of Hazardous Drug  Safe-Handling Precautions (PHDP) model (Lusk et al., 1997); The Chemotherapy Handling Questionnaire, developed by Polovich and Clark (2012), was used to measure chemotherapy handling practices. | | 27 | |
| **Ale Hashem & Baniasadi (2018) Iran** | | 80 | | To evaluate the knowledge, attitude, and practice (KAP) of oncology nurses towards the safe handling of Anti-neoplastic drugs. | | Knowledge, attitudes, and Safe handling practice | | Cross sectional study - six university hospitals | |  | | Six University Hospitals, 80 Nurses participated | Developed Questionaries: Demographic; Protocol knowledge & Standards; Attitudes towards working in oncology, concerns & feelings; direct handling practice. | | 26 | |  |
| **Asefa et al. (2021) Ethiopia** | | 77 | | To assess knowledge and practices of safe handling of CDs | | Knowledge and Safe Handling precautions, PPE usage | | Cross-sectional study | |  | | Nurses from one specialized hospital and one University Hospital | Adapted questionnaire. Variable: Knowledge; practice; Cytotoxic; Handling; Cytotoxic waste; Disposal waste; Personal Protective Equipment. | | 27 | |  |
| **Batista et al. (2021) Brazil** | | 35 | | To assess the knowledge of nursing professionals about the use of antineoplastic drugs in a general hospital. | | Knowledge, perceived risks, experience of side effects in handling CDs | | Descriptive and exploratory study with a qualitative approach | |  | | University Hospital | Semi-structured interview schedule | | 26 | |  |
| **Baykal et al. (2009) Turkey** | | 171 | | The purpose of determining the problems of nurses who work on oncology units and administer cytotoxic drugs, with their working conditions, personal rights and working life | | Working conditions, Perception of PPE usage, and contamination | | Analytic descriptive questionnaire. | |  | | Nine hospitals in Istanbul province nine (four private, two university and three public) hospitals | Local 47 -item: personal demographics (5); Working conditions (31 closed ended; 11 open; 12 classification). Expert validation only. Piloted in 15 nurses. | | 27 | |  |
| **Ben-Ami et al. (2001) Israel** | | 61 | | To examine the influence of the nurses’ beliefs, attitudes, and knowledge concerning occupational exposure, on their behavior and proper use of recommended protective measures. | | Framework – Phycological measures based on the Health Belief Model towards the Cytotoxic drugs and site observation of exposed and unexposed nurses. | | Descriptive questionnaire | | The Health Belief Model (HBM) and its extensive form | | Two Central Hospitals in Israel and community nurses | Demographic data: age, sex, marital status, State of birth, military service, education, number of years as an RN and as an oncology nurse. 2. Lifestyle, smoking, and other habits. 3. Personal and family medical, occupational, and exposure history. 4. General health beliefs and the nurses’ perceived personal health. 5. The nurses’ knowledge of proper use of the protective measures during preparation and administration of CDs, and knowledge of potential risks of CDs. 6. Health behavior and safe behavior while dealing with the CDs. 7. Psychological measures: health beliefs and attitudes towards the CDs according to the HBM components: 1. perceived susceptibility 2. perceived severity 3. perceived benefits 4. perceived barriers 5. self-efficacy | | 21 | |  |
| **Benoist et al. (2022) France** | | 64 | | To assess the perception, knowledge, and handling practices of all occupation level categories | | Knowledge and perceived risk of exposure to CDs | | Descriptive questionnaire study, performed through face-to-face interviews | |  | | University Hospital | Adapted questionnaire from Hon et al 2015. | | 27 | |  |
| **Bernabeu-Martinez. (2021) Spain** | | 65 | | To assess the perception of risk of exposure in the management of hazardous drugs (HDs) through home hospitalization and hospital units | | Knowledge, Perception of risk of exposure to HDs | | Cross sectional study, Questionnaire based | |  | | National questionnaire disseminated via email with electronic link. | Adapted Questionnaire twenty-one specific questions | | 22 | |  |
| **Borges et al. (2015) Brazil** | | 26 | | To implement best practices guidelines for minimizing chemical exposure risk of nurses in a chemotherapy unit (CTU) using the knowledge, attitudes, and practices survey (KAP) | | Adverse health effects, Knowledge, and perceived risk of exposure to CDs | | Quantitative, descriptive KAP questionnaire based. | |  | | Two cancer units in the National cancer institute | Developed KAP questionnaire to identify the knowledge, attitudes, and practices with respect to a specific topic. The Likert scale was utilized with the questions. | | 23 | |  |
| **Callahan et al. (2016) USA** | | 115 | | To identify factors associated with oncology nurses’ use of hazardous drug (HD) safe-handling precautions in inpatient clinical research units. | | Exposure knowledge, self-efficacy, barriers to personal protective equipment use, perceived risk, conflict of interest | | Descriptive, cross-sectional. | | Factors Predicting Use of Hazardous Drug (HD) Safe-Handling Precautions | | National Institutes of Health Clinical Centre in Bethesda, Maryland | | The Hazardous Drug Handling Questionnaire (HDHQ) developed by Polovich and Clark (2012) | 27 | |  |
| **Chen et al. (2016) Taiwan** | | 57 | | Aim of the study was to explore the concerns of nurses regarding their decision to use or not to use PPE in the cultural context of Taiwan. | | Ethnographic, Site Observation PPE usage, barriers of PPE usage, perception of risk, self- efficacy. | | Ethnographic Qualitative Study | |  | | Two Accredited medical centers with oncology team. | | Interviews and observation. Ethnographic interviews were conducted using 3 levels of questions: descriptive, structural, and contrasting. Descriptive questions provided the general features of the research phenomenon. | 24 | |  |
| **Colvin et al. (2016) USA** | | 33 | | The objective was to learn if current NIOSH PPE and hospital policy chemotherapy exposure controls were adhered to in actual clinical practice based on observation and nurses’ self-assessment. | | Site observation of safe handling measures and adherence to PPE | | Micro-ethnography and questionnaire | |  | | Large Cance Centre: Cleveland Clinic | | Observation and self-assessment of adherence; 15 item checklists on skills (NIOSH & ONS Guidelines & Institutional policies). | 26 | |  |
| **Constantinidis et al. (2011) Greece** | | 353 | | Aim of the study was to describe the existing knowledge and attitude of the healthcare workers regarding the risks concerning their work, as well as the adverse effects experienced by them in relation to their occupational exposure to chemotherapeutic agents | | Knowledge, safe handling, safety climate, side effects | | Questionnaire from twenty-four public and private hospitals covering the entire country between November 2006 and April 2007. | |  | | Questionnaire to twenty-four public and private hospitals covering the entire country | | The first part contained a series of demographic characteristics, working position information and smoking habits. The second part contained three groups of specific questions according to the type of exposure to the chemotherapeutic agents, that is, the transportation and storage, or the preparation and reconstitution procedure and finally administration and patient care. | 22 | |  |
| **Çınar, & Karadakovan (2022). Turkey** | | 117 | | To examine the risks faced by oncology nurses in the units they work in and occupational safety. | | Safe handling, Knowledge, safety climate | | Cross-sectional descriptive study | |  | | Questionnaire to cancer nurses actively registered  to the Oncology Nurses Association in Turkey | | Developed questionnaire. | 26 | |  |
| **Dejoy et al. (2017) USA** | | 1814 | | This study examined the effects of pertinent organizational safety practices and perceived safety climate on the use of personal protective equipment, engineering controls, and adverse events (spill/leak or skin contact) involving liquid antineoplastic drugs. | | Perceived risks, safe handling measures, and engineering controls for safe handling. | | Cross-sectional survey | |  | | National Web-based survey | | The survey included seven hazard modules and a core module in addition to a screening module. The data for this study came from the 2011 NIOSH Health and Safety Practices Survey of Healthcare Workers, an anonymous, multi-module, web-based survey. Details of survey is described in Steege et al,2014 | 19 | |  |
| **Graeve et al. (2017) ^a & b^ USA** | | 163 | | To develop and test a worksite intervention that protects healthcare workers who handle antineoplastic drugs from work-related exposures. | | Framework model variables, PPE usage, Surface contamination and workplace safety climate | | Intervention study. | | Factors Predicting Use of Hazardous Drug (HD) Safe-Handling Precautions | | A university hospital in a large midwestern metropolitan area and its outpatient chemotherapy infusion clinic. | | The Hazardous Drug Handling Questionnaire (HDHQ) developed by Polovich and Clark (2012) | 27 | |  |
| **Hanafi et al. (2017) Iran** | | 77 | | To examine all adverse effects associated with handling of antineoplastic drugs. | | Knowledge, Safe Handling measures, adverse effects, monitoring | | An observational cross-sectional survey  Mixed method- using observations and cross-sectional survey | |  | | Three tertiary care teaching hospitals in Tehran, Iran | | Questionnaire designed from recent guidelines. | 25 | |  |
| **He et al. (2017) USA** | | 467 | | To examine patterns and organizational correlates of personal protective equipment (PPE) use and hazardous drug spills. | | PPE use, safety climate and hazardous drug spills | | Cross-sectional mailed survey. | | Factors Predicting Use of Hazardous Drug (HD) Safe-Handling Precautions | | Oncology Nursing Society members who administer hazardous drugs. | | The Revised Hazardous Drug Handling Questionnaire measures frequency of using PPE Questionnaire. | 25 | |  |
| **Hon et al. (2015) Canada** | | 120 | | To explore the degree of contact with antineoplastics, knowledge of risks associated with antineoplastics, perceptions of personal risk, previous training with respect to antineoplastics, and safe work practices. | | Knowledge of risk, PPE usage, barriers, safe handling behavior | | Cross-sectional | |  | | Six acute care facilities in Vancouver, British Columbia. | | Self-administered questionnaire | 24 | |  |
| **Khan et al. (2012) Pakistan** | | 35 | | To measure the levels of nurse’s knowledge and attitude after the conduct of education session regarding chemotherapy administration and management. | | Knowledge, attitudes, training | | Single group pre-test post-test study design | |  | | Two oncology units of tertiary hospital, Pakistan | | Knowledge-11 questions; Attitude-21 items. The face, content, construct and criterion validity and reliability of questionnaire were established | 26 | |  |
| **Kim et al. (2019) South Korea** | | 872 | | To examine the safe handling practice of chemotherapeutic agents by Korean nurses working in inpatient units and to examine the relationship between Korean nurses’ perceptions of the hospital safety climate and adherence to the safety guidelines for handling chemotherapeutic agents. | | Perceived risks, safe handling measures, PPE | | A descriptive, correlational design with a cross-sectional survey | |  | | Cross-sectional survey using data from the Korea Nurses'  Health Study. | | The KNHS adopted the protocols and survey questions of the United States of America (U.S.). Nurses’ Health Study 3, with minor changes to reflect cultural and organizational differences | 27 | |  |
| **Kosgeroglu et al. (2005) Turkey** | | 121 | | The aim of this study was to determine both the level of information that nurses possessed and the method of administration nurses used during chemotherapeutic drug preparation and administration. | | Safe handling precautions, PPE usage, and site Observation | | Descriptive study  Mixed methods- Survey and site observations | |  | | The chemotherapy administration units of all hospitals in Eskisehir, west Turkey | | Twenty-five questions in the questionnaire along with observation questions 11 for self-care and 14 for environmental -OSHA Directives (1986) and related literature. | 27 | |  |
| **Kutlutürkan et al. (2022) Turkey** | | 80 | | The aim of this study was to determine oncology nurses’ views of the strengths, weaknesses, opportunities, and threats to oncology nurses. | | Knowledge, risks, and safe handling of awareness of CDs. The working conditions and emotions of nurses were also described through the SWOT method. | | Descriptive pilot study | |  | | The study population consisted of nurses who were members of the Oncology Nursing Association of Turkey | | Developed questionnaire based on a literature review and a SWOT template. | 27 | |  |
| **Kyprianou et al. (2010) Cyprus** | | 88 | | To evaluate the knowledge, attitudes, and beliefs of Cypriot nurses on their exposure to antineoplastic agents | | Knowledge, perception of risks of exposure, PPE usage, and side effects from exposure. | | A cross-sectional survey using a self- administered questionnaire | |  | | Nurses who work in three hospitals in Nicosia, Cyprus | | The questionnaire was originally compiled by Turk et al., to evaluate the knowledge, attitudes and safe behaviors of nurses’ handling cytotoxic drugs and was translated from Turkish to Greek by two bilingual volunteers. | 26 | |  |
| **Mahdy et al. (2017) Egypt** | | 65 | | To evaluate the effect of cytotoxic drugs safety guidelines on knowledge, safe handling practices and attitude of oncology nurses | | Knowledge, attitudes, Safe handling practices of CDs | | A Quasi experimental design was utilized to conduct this study. A quasi-experimental research design with one group pre-test, post-test was used to conduct this study. | | Factors Predicting Use of Hazardous Drug (HD) Safe-Handling Precautions | | Cancer center affiliated to Ain Shams University Hospitals, Cairo, Egypt. | | Questionnaire was developed by the researchers in an Arabic language based on the review of related literatures. The Hazardous Drug Handling Questionnaire (HDHQ) developed by Polovich and Clark (2012) | 27 | |  |
| **Nwagbo et al. (2017)**  **Nigeria** | | 100 | | To determine knowledge of chemotherapy and occupational safety measures of nurses in oncology units | | Knowledge, Side effects, Occupational safety measures related to CDs | | A cross sectional descriptive study | | Protection Motivation theory | | Cross‑sectional study among nurses in the oncology unit of University College Hospital | | Fifty-four item validated questionnaire; questionnaire was subjected to expert review to ensure content and face validity. | 27 | |  |
| **Orujlu et al. (2016) Iran** | | 54 | | To evaluate knowledge, attitude, and performance of oncology nurses and to survey nurses’ chemotherapy workload and the experienced side effects. | | Knowledge, attitude, safe handling measures, working conditions, and the experienced side effects. | | Quantitative Cross-sectional study | |  | | Four hospitals of Urmia University, Iran. | | Developed Questionnaires including The Hazardous Drug Handling Questionnaire (HDHQ) developed by Polovich and Clark (2012) | 23 | |  |
| **Polovich and Clark (2012) USA** | | 165 | | To examine relationships among factors affecting nurses’ use of hazardous drug (HD) safe handling precautions, identify factors that promote or interfere with HD precaution use, and determine managers’ perspectives on the use of HD safe-handling precautions. | | Knowledge, attitude, and safe handling measures. Framework analysis of the theoretical predictor variables. | | Cross-sectional, mixed methods | | Factors Predicting Use of Hazardous Drug (HD) Safe-Handling Precautions | | Mailed invitation to oncology centers across the  United States. | | The Hazardous Drug Handling Questionnaire (HDHQ) developed by Polovich and Clark (2012) | 27 | |  |
| **Shahrasbi et al. (2014) Iran** | | 225 | | To evaluate the attitude, knowledge and safe practices of nurses' handling cytotoxic drugs. | | Evaluation of exposure and contamination | | Quantitative Cross-sectional study and surface sampling and observation | |  | | Multiple sites, Nurses working in specialized cancer centers in Tehran | | Based on International Guidelines: American Society of health System Pharmacists; occupational safety and Health Administration; health and Safety Executive. Two parts: understanding of hazard & Reported side effects. | 21 | |  |
| **Silver et al. (2016) USA** | | 1094 | | To examine factors associated with adherence among 1094 hospital nurses who administered ADs. | | Engineering controls, work practices, nurse perceptions, use of personal protective equipment PPE | | Quantitative Cross-sectional study | |  | | National Survey- The study population primarily included members of professional practice organizations representing health care occupations which routinely use, or encounter selected chemical agents. | | Adapted Questionnaire included seven hazard modules and a core module in addition to a screening module. | 25 | |  |
| **Simons & Toland (2017) UK** | | 55 | | to explore the immediate adverse effects experienced by nurses during the administration of systemic anti-cancer therapy (SACT), specifically cytotoxic chemotherapy, and whether closed systems are being used to minimize exposure risk | | Side effects, usage of closed systems | | Cross sectional survey | |  | | Study population selected from local network of nurses and survey sent through social media of the nurses’ network. | | The questions were compiled by the authors based on available research and guidelines that list the potential adverse effects of occupational exposure to cytotoxic chemotherapy drugs | 19 | |  |
| **Simons & Toland (2019) UK** | | 61 | | To explore awareness, knowledge, training, and use of protection measures by healthcare personnel working in areas where Systemic anti-cancer treatment is administered. | | Knowledge of risks, PPE usage, and Engineering control | | Quantitative Cross-sectional study | |  | | aimed at healthcare personnel working in SACT administration areas. | | Certain Questions included in the survey were adherence to training and annual update from (Control of Substances Hazardous to Health (COSHH) 2002, HSE 2014, Santillo et al 2018) | 24 | |  |
| **Soheili et al. (2021^a, b & c^) Iran** | | 52 | | Exploring oncology nurses’ perceptions regarding work- related stressors, health work environment & occupational needs. | | Knowledge of risks, PPE usage, and Engineering control | | Qualitative descriptive study. | |  | | eight cancer treatment centers in different cities of Iran | | In-depth semi-structured interviews. Each interview started with a general question and continued with specific questions in line with the objectives of the study | 27 | |  |
| **Srisintorn et al. (2021)**  **Thailand** | | 884 | | aimed to evaluate the level of PPE usage and factors predicting PPE usage among nurses and nurse assistants in Thailand. | | Knowledge, attitude, and safe handling measures. Framework analysis of the theoretical predictor variables. | | Quantitative Cross-sectional study | | Factors Predicting Use of Hazardous Drug (HD) Safe-Handling Precautions | | survey was conducted in a university hospital and two general hospitals | | The Hazardous Drug Handling Questionnaire (HDHQ) developed by Polovich and Clark (2012) | 26 | |  |
| **Topcu et al. (2017)**  **Turkey** | | 15 | | Aimed towards describing attitudes, opinions and experiences of nurses administering these drugs about safe handling precautions. | | Evaluate factors that affect nurses using safe handling. | | Qualitative study design. | | Health Belief Model (HBM) | | The study was conducted in two hospitals | | A semi-structured interview including open-ended questions and based on HBM was used for data collection. Questions were based on the following constructs: perceived benefits of safe handling precautions, perceived barriers in taking safe handling precautions, perceived seriousness for safe handling precautions, perceived sensitivity for safe handling precautions, cues to action for safe handling precautions. | 19 | |  |
| **Tuna & Baykal (2017)**  **Turkey** | | 25 | | To determine the working conditions of the oncology nurses in terms of employee safety as well as their knowledge levels regarding the safe use of antineoplastic drugs. | | Safe handling, side effects, working conditions, training, and knowledge. | | Qualitative study with phenomenological design. | |  | | Oncology nurses who were working in an oncology center in the city of Istanbul | | Qualitative study conducted in the phenomenological design. | 26 | |  |
| **Turk et al. (2004) Turkey** | | 120 | | To evaluate the level of knowledge of nurses on the health effects and the routes of exposure to CDs, to clarify the protective measures while handling these agents and to determine the influence of this knowledge on clinical attitudes, behavior, and actual usage of safety measures. | | Knowledge, perception of risk, safe handling precautions, and PPE usage. | | Analytic cross sectional study Mixed methods- Direct observations and survey | |  | | Cancer nurses at university teaching hospital | | Two self-reported questionnaires were used to collect the data of the study | 24 | |  |
| **Verity et al.**  **(2008) UK** | | 257 | | Aim of this study was to describe nurses’ experiences, attitudes, and educational preparation for the chemotherapy administration process. | | Experiences, attitudes towards and educational preparation for administrating chemotherapy and determine factors that influence their perceptions. | | Postal survey | |  | | A postal survey was conducted across twenty-six London hospitals providing cancer services. | | Developed Questionnaire: Education, Worries and Attitudes Questionnaire–Hospital Version. | 27 | |  |

Supplementary material: The main characteristics of the studies
